# Dated Plant Phylogenies Resolve Neogene Climate and Landscape Evolution in the Cape Floristic Region

**DOI:** 10.1371/journal.pone.0137847

**Published:** 2015-09-30

**Authors:** Vera Hoffmann, G. Anthony Verboom, Fenton P. D. Cotterill

**Affiliations:** 1 Department of Biological Sciences, University of Cape Town, Cape Town, South Africa; 2 Africa Earth Observatory Network (AEON), Geoecodynamics Research Hub, Department of Botany and Zoology, University of Stellenbosch, Stellenbosch, South Africa; University of Colorado, UNITED STATES

## Abstract

In the context of molecularly-dated phylogenies, inferences informed by ancestral habitat reconstruction can yield valuable insights into the origins of biomes, palaeoenvironments and landforms. In this paper, we use dated phylogenies of 12 plant clades from the Cape Floristic Region (CFR) in southern Africa to test hypotheses of Neogene climatic and geomorphic evolution. Our combined dataset for the CFR strengthens and refines previous palaeoenvironmental reconstructions based on a sparse, mostly offshore fossil record. Our reconstructions show remarkable consistency across all 12 clades with regard to both the types of environments identified as ancestral, and the timing of shifts to alternative conditions. They reveal that Early Miocene land surfaces of the CFR were wetter than at present and were dominated by quartzitic substrata. These conditions continue to characterize the higher-elevation settings of the Cape Fold Belt, where they have fostered the persistence of ancient fynbos lineages. The Middle Miocene (13–17 Ma) saw the development of perennial to weakly-seasonal arid conditions, with the strongly seasonal rainfall regime of the west coast arising ~6.5–8 Ma. Although the Late Miocene may have seen some exposure of the underlying shale substrata, the present-day substrate diversity of the CFR lowlands was shaped by Pliocene-Pleistocene events. Particularly important was renewed erosion, following the post-African II uplift episode, and the reworking of sediments on the coastal platform as a consequence of marine transgressions and tectonic uplift. These changes facilitated adaptive radiations in some, but not all, lineages studied.

## Introduction

A robust knowledge of the palaeoenvironment underpins both our understanding of contemporary biodiversity [1,2] and our ability to manage and conserve biodiversity in the face of ongoing environmental change [3]. Invariably, however, palaeoenvironmental inference relies on palaeontological (e.g. macro- and microfossils; [4,5]) and abiotic proxies (e.g. δ^18^O; [6]) whose spatial and temporal resolution is often limited, especially in relation to phenomena taking place at local and regional scales. Insights gained from the fossil record, for example, are commonly fragmentary owing to spatiotemporal variability in taphonomic conditions [7]. In addition, many fossil deposits contain material sourced from extensive areas (e.g. oceanic pollen cores; [8]), which obscures the spatial resolution of source conditions. Abiotic proxies present challenges of their own. Some are difficult to interpret because they are controlled by multiple factors (e.g. δ^13^C; [9]), while others record global-scale phenomena (e.g. δ^18^O records global temperature fluctuations; [10]), thereby limiting their utility to resolve events at more local scales. Finally, it is not uncommon for different proxies to yield contrasting interpretations, exacerbating uncertainty of palaeoenvironmental reconstructions [11].

These challenges are exemplified in our palaeoenvironmental understanding of the Cape Floristic Region (CFR; [12]), an area renowned for its remarkable floristic richness and endemism. Although climatic and geological events during the Neogene are commonly invoked to explain the contemporary richness of the CFR flora [13–15], a lack of certainty about the extent and timing of these events hampers robust testing of these ideas. For example, the development of the modern summer-arid climate of the CFR has frequently been linked to initiation of oceanic forcing by upwelling of the Benguela current 10–14 Ma [16], as inferred on the basis of isotopic evidence for Antarctic ice sheet expansion [10]. The records contained in the offshore deposition of sediment, nanofossils [17,18] and marine illite, however, present a more complex picture of climatic shifts across the region. These data imply a prevalence of arid conditions along the CFR west coast from 11.6 to 7.7 Ma and again from 2.8 Ma to the present, the intervening period being interpreted as comparatively mesic [19]. The latter aridification episode appears to have been dramatic, coinciding with a sharp decline in sea surface temperatures (inferred from alkenone proxies [20]) and its timing is closely congruent with the presumed formation of a tectonically-induced, western summer-rain shadow ~5 Ma [21–23]. Based on these patterns, it has recently been postulated that the modern seasonally-arid climate of the western CFR, and its associated flora, was only established well after the initiation of Benguela upwelling, perhaps as recently as 3–5 Ma [24].

Although the first appearance of arid-adapted elements in the fossil record offers a means to constrain the inferred origination time of the modern summer-arid climate of the western CFR, a severe dearth of Neogene fossil sites, particularly in the interior, is critically limiting. Only one onshore site, at Langebaanweg, has been dated confidently to the Miocene-Pliocene boundary, and the patterns there are difficult to interpret. While the presence of a mesic Pliocene flora at Langebaanweg, including palms, suggests a delayed onset of seasonal aridity [25,26], the estuarine/riparian setting of this site raises questions over its representativeness of inland palaeoenvironments. In this context, a near-shore pollen record of Orange River deltaic sediments [8] may provide a better indication of when an arid-adapted flora emerged in the western CFR. This record suggests the replacement of subtropical thicket elements by Cape floristic elements ~6–10 Ma, with arid-adapted Aizoaceae first appearing ~8 Ma. Unfortunately the spatial context of these changes remains imprecise owing to the large terrestrial source area integrated into these marine sediments [8]. An onset of seasonally-arid conditions ~7–8 Ma is, however, consistent with isotopic signatures obtained from fossil ratite egg shells sampled from southern Namibia [27].

A paucity of direct geological evidence renders our understanding of CFR landscape evolution through the Neogene even less certain than our understanding of palaeoclimates. The traditional hypothesis, that the CFR was geologically and geomorphologically stable through much of the Cenozoic [28,29], has some support, at least for the uplands. Recent cosmogenic dates on river terraces [30] and elevated pediments [31] testify to the remarkable durability of the quartzitic rocks which form the backbone of the Cape Fold Mountains; so the rugged topography of the latter is arguably a legacy of coastline erosion initiated by the Cretaceous separation of the Falkland and African plates. At lower elevations, however, proposed Early Pliocene uplift along the Ciskei-Transkei flexure axis probably stimulated renewed erosion [22,32], resulting in widespread removal of a silcrete duricrust layer which had previously capped the Cape lowlands, which then exposed the clay-rich, shale- and granite-derived substrata that characterize these environments today [33]. The implication is that the CFR lowlands, unlike the uplands, may have been a focus of considerable geomorphological activity through the Neogene. A critical assessment of this idea is, however, hampered by a lack of direct geological evidence, such as that provided by low temperature thermochronology [34], and ideally requires precise dates on landforms at fine spatial scales.

Faced with these uncertainties and data gaps, the extant biota of the CFR offers a way forward. In the context of molecularly-dated phylogenies of species-rich clades, ancestral reconstruction of habitat variables [35] yields insights into the locations and timings of particular habitat shifts. Dated nodes in these phylogenies can potentially indicate, or at least constrain, the times at which these habitats first arose [36]. Crucially, congruence in the timing of specific habitat shifts across multiple lineages distinguish between, on the one hand, shifts attributable to broad-scale palaeoenvironmental change, and, on the other, shifts reflecting idiosyncratic, lineage-specific dispersals between pre-existing habitats. This is because broad-scale palaeoenvironmental changes are likely to elicit common adaptive responses in multiple affected lineages, preserved as a signature of synchronous habitat shifts across co-occurring clades. The spatiotemporal resolution associated with habitat mapping obviously depends on the robustness and precision of the underlying molecular clock calibrations and, equally, the degree of stenotopy (and thus habitat fidelity) of individual species in relation to the habitat parameters under investigation. In the context of robust, precise date estimates and pronounced habitat fidelity, however, this approach promises spatiotemporal resolution exceeding that of traditional proxies. To date, the use of replicated lineage histories as records of palaeoenvironmental change, recently termed the ‘geoecodynamic approach’ [37], has been employed in a diversity of systems to gain insights into biome history [38,39], drainage evolution [40,41], episodes of orogenesis [42], palaeoclimatic changes [43] and the evolution of historical fire regimes [36,44]. Although habitat mapping has been employed to constrain timings of habitat shifts within individual lineages of Cape plants [44–47], its utility as an indicator of broad-scale palaeoenvironmental change remains underexploited.

Here we assemble environmental data for 533 plant species, comprising 5.6% of the angiosperm flora of the CFR and representing 12 prominent clades. We then reconstruct the evolutionary histories of these lineages and use our reconstructions to trace major palaeoenvironmental changes through the Neogene and Quaternary. Our analyses are directed towards three objectives. First, by testing whether associations with aseasonal climates and/or quartzitic substrata are consistently recovered as ancestral, we evaluate the hypothesis that these conditions were prevalent in Early- to Late-Miocene environments of the CFR. Second, by mapping habitat associations on dated phylogenetic trees, we evaluate whether occupation of the modern summer-arid climate of the western CFR coincided with the initiation of Benguela Upwelling, and whether the occupation of non-quartzitic substrata on the Cape lowlands was linked to Pliocene uplift. In addressing this question, we account for the possibility that occupation of non-quartzitic substrata was prompted by climatically-forced contraction of a pre-existing thicket/forest flora [48,49] rather than geomorphological change *per se*. Finally, we test whether the major palaeoenvironmental changes documented here precipitated accelerated diversification in the lineages under study. The appearance of arid or seasonally-arid conditions in the western CFR is commonly suggested to have stimulated floristic radiation, principally through its creation of novel ecological opportunities [14,46,50], and a similar role has recently been hypothesized for geomorphological controls [33].

## Materials and Methods

### Lineage sampling

Selection of study lineages (Table 1) was done at the start of the study, in early 2010. Guiding criteria were: (i) the desire to capture a representative phylogenetic spread of groups; (ii) the availability of phylogenetic sequence data, with >70% of species sampled; (iii) a significant proportion of species being Cape Floristic Region (CFR)-native; (iv) evidence of interspecific variability in substrate and climatic niche preferences; (v) the availability of, or the possibility of generating, spatially-precise specimen locality data for all species; and (vi) evidence to show that the history of the lineage spanned as much of the Neogene as possible. A total of 12 lineages were sampled, representing six families of flowering plants. These are: Arctotidinae [51] and the *Stoebe* alliance [52,53] (Asteraceae); *Moraea* [45] (Iridaceae); *Pterygodium*, *Disperis* [54] and *Satyrium* [55] (Orchidaceae); *Ehrharta* [46], *Pentameris* and *Tribolium* [56] (Poaceae); *Leucadendron* [57] and *Protea* [58] (Proteaceae); and *Elegia-Thamnochortus* [59] (Restionaceae).

**Table 1:** List of the phylogenetic datasets included in this study.

Family	Clade	Sampling density	Marker		Model	Source
			Nuclear	Plastid		
Asteraceae	Arctotidinae	65%	ITS		GTR + Γ	[51]
				psbA-trnH	GTR + Γ	
				trnT-trnLF	GTR + Γ	
	*Stoebe*	96%	ITS		GTR + Γ	[52,53]
			ETS		HKY + I + Γ	
				trnLF	F81	
				trnTL	GTR	
				psbA-trnH	GTR + Γ	
Iridaceae	*Moraea*	82%		trnLF	GTR + Γ	[45]
				rps16	GTR + I + Γ	
				rbcL	GTR + I + Γ	
Orchidaceae	*Disperis*	83%	ITS		GTR + I + Γ	[54]
	*Pterygodium*	83%				
				matK	GTR + I + Γ	
				trnLF	GTR + Γ	
	*Satyrium*	93%	ITS		GTR + Γ	[55]
				matK	GTR + I + Γ	
				trnLF	GTR + Γ	
				trnL	GTR + I	
				trnSG	GTR + Γ	
Poaceae	*Pentameris*	87%	ITS		GTR + I + Γ	[56]
	*Tribolium*	93%		trnLF	GTR + I + Γ	
				rpl16	GTR + I + Γ	
				atpB-rbcL	GTR + Γ	
	*Ehrharta*	85%	ITS		GTR + Γ	[46]
				trnLF	GTR + Γ	
Restionaceae	*Elegia-Thamnochortus*	90%		trnK-matK	GTR + I + Γ	[59]
				atpB-rbcL	GTR + I + Γ	
				trnLF	GTR + I + Γ	
Proteaceae	*Leucadendron*	73%	ITS		GTR + I + Γ	[57]
	*Protea*	98%	ITS		GTR + I + Γ	[58]
			ncpGS		GTR + Γ	
				atpB-rbcL	HKY + Γ	
				rps16	GTR + Γ	
				trnLF	GTR + I + Γ	

Sampling density, calculated as a percentage of species listed for the CFR by [12], was determined by the availability of both DNA sequence and usable geospatial data. Also indicated are the gene regions analysed, and the substitution models determined as optimal by MrModeltest 2.3. The source publication for each data set is shown.

### Phylogenetic inference and molecular dating

Phylogenetic inference and molecular dating were performed in BEAST v1.5.3 [60]. Since reliable fossil evidence was not available for the clades sampled [21], species-level phylogenies were calibrated by applying secondary calibrations obtained from fossil-calibrated higher-level phylogenies. Hence, a two-step approach was used to date these lineages. The first step involved calibrating a set of higher-level phylogenies, using published alignments (Table 2). In cases where detailed dating analyses existed for the higher-level groups, these were evaluated in terms of the placement and validity of the fossils, and subsequently re-run in BEAST, either omitting or including selected fossils.

**Table 2:** Phylogenetic datasets and associated fossil calibrations used to determine secondary calibration points for the species-level phylogenies.

Family (Source)	Node	Age (Ma)	Fossil	Description	Source
Orchidaceae [60]	A	15–20	*Meliorchis caribea*	Stem of Goodyerinae	[68]
	B	20–23	*Dendrobium winikaphyllum*	Split *Dendrobium*—*Bulbosa*	[69]
	C	20–23	*Earina fouldenensis*	Split *Earina*—*Agrostophyllum*	[69]
Poales [62]	A	≥70	*Milfordia* pollen	Stem node of Restionaceae	[74]
	B	≥70	*Monoporites* pollen	Stem node of Poaceae	[74,76]
	C	≥60	Cyperaceae fruit	Stem node of Cyperaceae	[78]
	D	≥60	Restionaceae pollen	Crown node of Restionaceae s.s.	[73,79]
	E	≥55	Multiflowered grass spikelet	Stem node of BEP-PACCAD	[75,77]
	F	≥40	Mapanioid fossil sedge	Stem node of Mapanioideae	[83]
	G	≥35	North American phytoliths	Split BEP-PACCAD	[80]
	H	≥19	Chloridoid phytoliths	Stem node of Chloridoideae	[72]
Proteaceae [66]				Analysis not repeated.	
Asteraceae [53]				Analysis not repeated.	
Asparagales [67]				Analysis not repeated	

Column 2 provides the reference code used for each calibration node (as shown in Figures A and B in S1 File), column 3 the corresponding age constraint, column 4 a description of the fossil used, and column 5 a description of the calibration node. Finally, column 6 indicates the source reference(s) used to determine the age constraint. The BEP clade comprises Bambusoideae, Ehrhartoideae, Pooideae, and the PACCAD clade comprises Panicoideae, Arundinoideae, Chloridoideae, Centothecoideae, Aristidoideae, Danthonioideae [77].

#### Higher-level dating analyses

Following arguments presented in the literature [61,62], fossil calibrations were assigned to the stem rather than the crown nodes of the clades which they represent. Since fossils impose a minimum constraint on the ages of lineages, priors on fossil-calibrated nodes were set as lognormal, as recommended by [63], with the (log) mean of the lognormal distribution specified such that the median equalled the estimated minimum age of the fossil. This allows for the node to be slightly younger (accommodating error in fossil age estimation) but considerably older than the reference fossil, thereby accommodating fossil age error and the possible existence of older, as yet undiscovered fossils. Given that single fossils do not provide an indication of the shape of the lognormal distribution, the (log) standard deviations on priors were set arbitrarily to 50% of the mean value, such that the 95% confidence interval (CI) spanned a realistic time period. As a general rule, the zero offset was set to 0.9 times the mean. For most nodes, this gave very reasonable priors. For younger nodes, however, this procedure yielded 95% CIs that were often too narrow to be realistic or to cover the full range of possible ages for the relevant fossil. For nodes younger than 20 Myr, therefore, the zero offset was set to 0.8 times the mean.

BEAST analyses employed a mixed-model approach, with different substitution models applied to each gene region. Model selection was done under the Akaike Information Criterion (AIC) using MrModeltest v2.3 [64]. Tree priors were defined using a Yule process, and the default coalescent process implemented in BEAST was used to obtain starting trees. In some instances, however, the imposition of hard age constraints rendered the use of such starting trees unworkable and for these analyses randomly resolved starting trees with imposed calibration constraints were used. Xml files were assembled in BEAUTi v1.5.3 [65]. Both higher-level analyses comprised three independent runs, either of 5 x10^7^ (Orchidaceae) or 10^8^ (Poales) generations, sampling every 5,000 and 10,000 generations, respectively. Tracer v1.5 [60] was used to check for convergence across runs, and to determine appropriate burn-ins. For both analyses, the first 1,000 trees in each run, equivalent to a burn-in of 10% (or 5 x10^6^ generations for Orchidaceae and 10^7^ generations for Poales), were discarded prior to calculation of the maximum clade credibility (MCC) tree using TreeAnnotator v1.5.3 [65].

For Proteaceae [66], Asteraceae [53] and Asparagales [67] we made use of existing higher-level dating analyses, since the fossil placement procedures and dating methodology (BEAST) used in those studies were comparable to the approach adopted here. For Arctotidinae, *Leucadendron*, *Moraea*, *Protea*, and *Stoebe*, therefore, secondary calibrations were extracted directly from these chronograms (Table 2). In order to derive secondary calibrations for Coryciinae (which includes *Disperis* and *Pterygodium*) and *Satyrium*, secondary calibrations were obtained by applying BEAST-dating to a 61-taxon matrix of Orchidaceae [68] to which 23 further taxa were added to ensure the inclusion of appropriate secondary calibration nodes. This analysis was calibrated using the same three fossils [68,69] as were used previously by Gustafsson *et al*. [70] (Table 2). In contrast to Gustafsson *et al*. [70], however, we assigned these fossils to stem nodes as opposed to crown nodes. In a similar manner, secondary calibrations for *Ehrharta* and Danthonieae (which includes *Pentameris* and *Tribolium*) were obtained by applying BEAST-dating to the Poales matrix of Christin *et al*. [71]. Calibration of this analysis was achieved using a selection of fossils [72–80] employed in previous dating analyses [78,79,81,82], plus a recently described mapanioid sedge fossil [83]. To facilitate computation, the Poales matrix was pared down to a subset of 90 of the original 338 taxa, with fifteen new taxa being added to ensure the inclusion of appropriate secondary calibration nodes.

#### Species-level dating analyses

Species-level dating analyses were run using published alignments whose completeness of species sampling ranged between 65% and 98% (Table B in S1 File). In the case of *Pentameris* and *Tribolium*, dates were obtained via a single dating analysis of Danthonieae, while for *Disperis* and *Pterygodium*, a single dating analysis of Coryciinae was employed. Since the species-level analyses were all secondarily calibrated, the age priors on calibration nodes were specified as normal, with their means and 95% CIs set to match the medians and 95% HPDs of the posterior distributions obtained from the higher-level dating analyses (Table 2). In other respects, however, the species-level analyses were conducted in an identical manner to the higher-level analyses, each analysis comprising three runs of 5 x 10^7^ generations, with samples drawn every 5,000 generations.

### Selection and scoring of environmental variables

For palaeoenvironmental reconstruction, all Cape species included in the 12 dated phylogenies were scored for: (i) monthly precipitation coefficient of variation (PCV); (ii) precipitation in the driest quarter of the year (PDQ); and (iii) geological substratum. This was achieved by geo-referencing specimen record data from the Bolus (BOL), Pretoria (PRE) and Compton (NBG) herbaria as precisely as possible, and using suitably precise records to query relevant environmental layers within a GIS framework (see Table 3), the latter step being analysed using ArcGIS v9.3 in conjunction with Hawth’s Analysis Tools [84]. For habitat characterization, we only made use of records having a geo-reference precision <2,000 m, this threshold being determined as a trade-off between precision and the number of specimens sampled per species. Following data cleaning and removal of duplicate records, the total number of records used for habitat scoring varied from 1 to 250 records per taxon.

**Table 3:** Description, delimitation and spatial extent of habitat states.

Variable	Description	Area (km^2^) and percentage of total CFR area (parentheses)	Percentage overlap		Resolution (Source)
			with PDQ <75mm/PDQ >75 mm	with PCV <60%/PCV >60%	
Coefficient of variation of monthly precipitation (PCV)					~ 1 km [86]
PCV <60%	Precipitation aseasonal to weakly seasonal	86,242 (73.6)	52.1/47.9	-	
PCV >60%	Precipitation strongly seasonal	30,988 (26.4)	98.1/1.9	-	
Precipitation in the driest quarter (PDQ)					~ 1 km [86]
PDQ <75 mm	Driest quarter strongly arid	75,335 (64.3)	-	59.6/40.4	
PDQ >75 mm	Driest quarter weakly-arid to mesic	41,895 (35.7)	-	98.6/1.4	
Substratum					1:250,000
Quartzite	Sandstones and arenites of the Table Mountain and Witteberg Groups.	43,903 (37.5)	51.4/48.6	76.9/23.1	(Geoscience Council, SA)
Shale	Shales and mudrocks of the Bokkeveld, Cederberg, and Pakhuis Formations.	42,572 (36.3)	72.5/27.5	84.2/15.8	
Granite	Granites of Cape Granite Suite.	1,745 (1.5)	57.9/42.1	58.9/41.1	
Alluvial	Alluvial deposits on mountains and along rivers.	8,479 (7.2)	80.1/19.9	74.5/25.5	
Lowland sands	Acidic clays and sands, especially on the western coastal plain.	7,917 (6.8)	95.8/4.2	16.6/83.4	
Calcareous	Calcretes and calcarenites. Mostly coastal.	8,149 (7.0)	54.6/45.4	61.6/38.4	

The spatial extent (area) of each state is expressed in approximate km^2^ (column 3), and as a percentage of the total area of the CFR (in parentheses). Spatial overlaps with states of the two climatic variables are also indicated (columns 4 and 5). Column 5 indicates the resolution of the GIS layers used, along with sources (in parentheses).

Although the first two variables are continuous, we elected to score them in a binary manner and reconstruct them using discrete character state reconstruction methods. We did this in order to circumvent limitations of continuous trait reconstruction methods, which have been shown to yield very different reconstructions depending on the choice of method [85]. Of particular concern in this study, is the fact that the most widely used continuous trait reconstruction methods (squared-change parsimony and model-based methods) smooth change across all branches in the tree [85] and, in so doing, produce ancestral values which are weighted averages of the tip values. These methods are therefore unlikely to reconstruct ‘extreme’ values (i.e. corresponding to the smallest or largest tip value) on deeper ancestral nodes, with ancestral values outside the tip range being impossible. This limitation is particularly problematic in the context of a changing environment in which extreme ancestral trait values are to be expected. Binary scoring circumvents this problem, to some extent, by not specifying outer limits on the states defined and so allowing ancestors to assume extreme values (e.g. PDQ <75 mm or PDQ >75 mm).

#### Coefficient of variation of monthly precipitation

**Fig 1:**
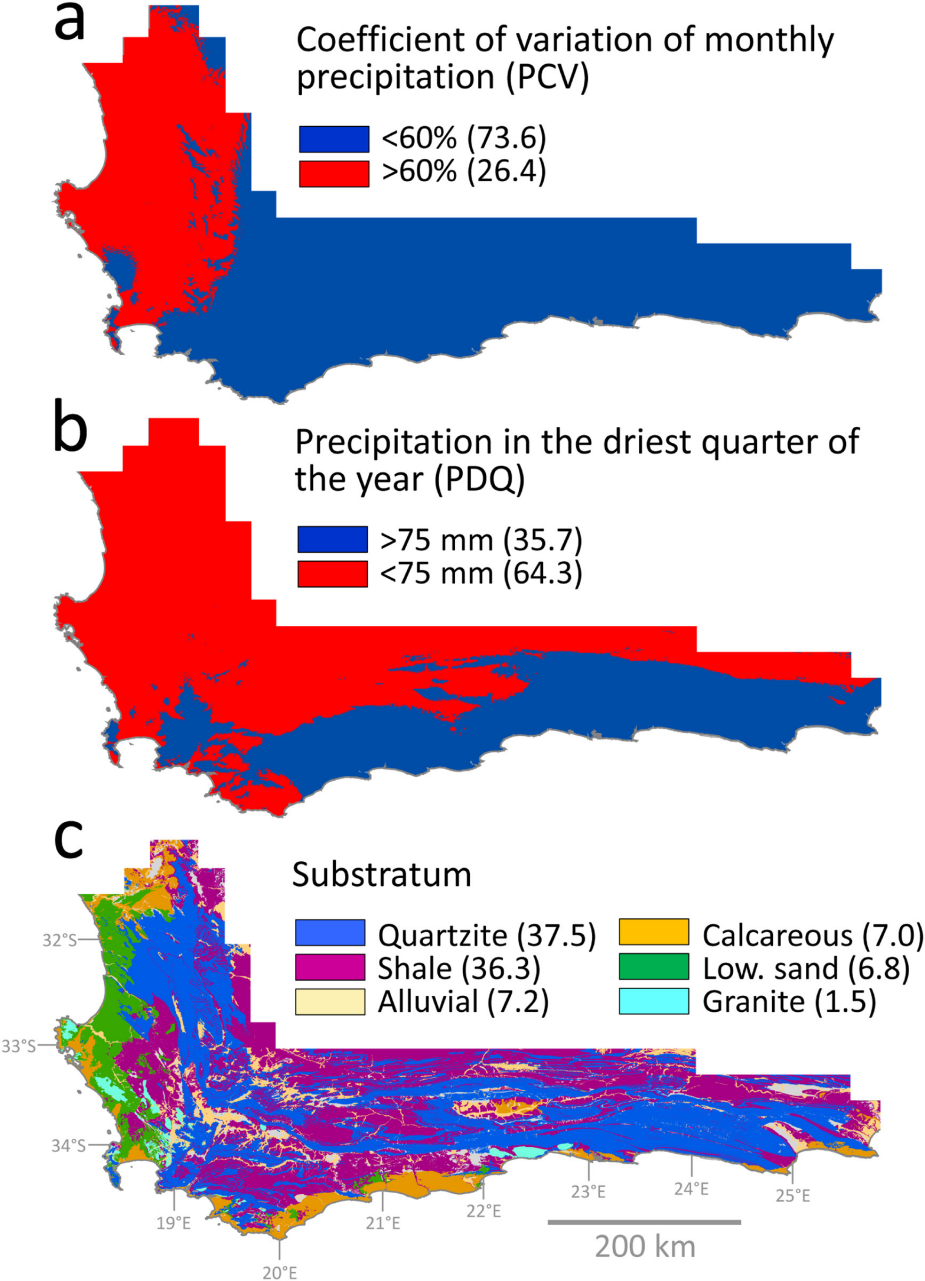
Environmental measures of the Cape Floristic Region (CFR) utilized in the study for delineating habitat occupation. Maps of the CFR depicting spatial variation in: a) coefficient of variation of monthly precipitation (PCV); (b) precipitation during the driest quarter of the year (PDQ); and c) substratum (bedrock geology). The percentage area covered by each habitat category is indicated in parentheses. For PCV, a value >60% indicates a strongly seasonal rainfall regime, with the bulk of precipitation falling within the winter months.

Rainfall seasonality, expressed as the coefficient of variation of monthly precipitation (PCV), was scored using the appropriate 30 arc-second (~1 km) resolution layer in the WorldClim Global Climate database ([86]; BioClim variable 15). This variable, which measures the evenness of rainfall through the year, attains high values under conditions of strong precipitation seasonality. Following Chase and Meadows [87] the boundary between aseasonal and seasonal climate was defined by a seasonality threshold of 60%, with PCV <60% being defined as aseasonal and PCV >60% as seasonal. This threshold identifies all of the eastern CFR and the high-elevation zones of the west as aseasonal (Fig 1a).

#### Precipitation in the driest quarter

Precipitation in the driest quarter (PDQ) was scored using the appropriate 30 arc-second (~1 km) resolution layer in the WorldClim Global Climate database ([86]; BioClim variable 17). Since this variable quantifies the amount of precipitation during the driest three months, it reflects the intensity of drought at the driest time of the year, rather than being a measure of moisture seasonality. As such, it is more a measure of general aridity than of moisture seasonality. We coded this variable using a threshold of 75 mm (i.e. PDQ <75 mm or PDQ>75 mm), which identifies aridity as widespread in the CFR, being absent only from the southeast and the higher-elevation zones of the west and the interior (Fig 1b).

#### Substratum type

Species substrate associations were characterized using a 1:250,000 layer produced by the South African Council for Geoscience, which classifies the geology of the Cape into approximately 130 different lithologies. We grouped these lithologies into six broad substrate categories (quartzitic sandstone, shale, granite, calcareous substrata, alluvial deposits and lowland sands) which correspond to those traditionally considered biologically relevant [88]. Matching earlier accounts, our data identify most of these substrate classes as being widely, though often patchily, distributed in the CFR (Fig 1c).

To accommodate limitations in the resolution of the layers used to score species’ habitats, and to allow for possible specimen misidentifications, outlier specimens were ignored when scoring species’ habitat associations. In the case of the two continuous variables, this was achieved by ignoring specimens whose values fell outside the 25–75% quartile range. Where this range fell entirely within one of the binary categories, a species was scored as monomorphic. On the other hand, where a species’ range spanned multiple categories, it was scored as polymorphic. The use of ranges in scoring habitats is preferable to the use of mean or median values, as the latter do not represent the full range of habitats occupied [35]. In the case of substrate type, a species was scored as monomorphic if ≥80% of specimens were identified as occurring on a single substratum. Where this was not the case, the species was scored as polymorphic with respect to the highest-frequency states which cumulatively accounted for ≥80% of the specimens sampled.

### Ancestral habitat reconstructions

Ancestral habitat associations were inferred using the program Lagrange v20120508 [89], which evaluates the likelihoods of alternative reconstructions in the context of a continuous-time model dispersal- extinction-cladogenesis model. Although this method was developed explicitly for the purpose of inferring geographic range shifts, the effectiveness of niche conservatism in limiting dispersal between habitats renders it appropriate for ancestral habitat inference. We preferred this method over standard ancestral character state procedures because, although the current implementation offers limited scope for incorporating phylogenetic uncertainty, it has the significant benefit of allowing habitat polymorphisms to be explicitly reconstructed at ancestral nodes, in the context of a defined model-based framework. While Lagrange enables the user to impose constraints relating to both the ranges and the dispersal patterns allowed [90,91], no such constraints were imposed here because they were not justifiable a priori. Lagrange reconstructions were performed using the MCC trees generated in BEAST. Outgroups and extra-CFR species were pruned out prior to ancestral habitat inference because some of the habitat variables (e.g. substratum type, precipitation seasonality) acquire a different biological meaning and/or cannot be comparably scored outside the CFR. Omitting these taxa effectively treats them as unknown with respect to these variables.

For the purpose of mapping state shifts (Figures M-O in S1 File), the probability of a state (representing either a monomorphism or a particular polymorphism) on a branch was determined as the sum of the probabilities of the state on that branch across all alternative Lagrange reconstructions. For the purpose of our interpretations, we treated a state probability ≥0.55 as constituting support for a particular reconstruction, and where no single state had a probability ≥0.55 the reconstruction was treated as uncertain. Thus we considered a transition to a particular habitat state (monomorphism or polymorphism) to have taken place where the probability of that state on a branch exceeded 0.55, and where the preceding branch either had an alternative habitat state or was uncertain.

To assess the sensitivity of our results to the particular support threshold used (≥0.55), we compared the inferred timing of the earliest occupation of each habitat by each lineage, under support thresholds of both ≥0.55 and ≥0.70 (as indicated in Figures M-O in S1 File). This comparison revealed that increasing the support threshold to ≥0.70 altered the inferred timing of only 14 “earliest” habitat shifts, consistently moving these later. Since these changes were generally small in magnitude (mean ± s.d. = 1.91 ± 1.39 Myr), the average earliest occupation of each habitat, determined across all study lineages, was not greatly affected (moved forward by 0.06–0.68 Myr, or 0–5.9%), representing a mean (± s.d.) time shift of 0.27 ± 0.23 Myr (3.0 ± 2.4%). In addition, these adjustments rarely affected the temporal sequence of earliest habitat shifts within a lineage. We conclude that our results are relatively robust to the choice of support threshold.

### Tests of association

To test whether the occupation of seasonal environments was associated with initiation of Benguela upwelling in the Middle to Late Miocene, or with tectonic uplift at the Miocene-Pliocene boundary, one-sample t-tests were used to assess whether the mean time of first appearance of seasonal endemics in each lineage differed significantly from 5 or 10 Ma. One-sample t-tests were also used to evaluate whether or not the first appearances of shale- and calcareous-endemics coincided with tectonic uplift at 5 Ma. Paired-sample t-tests or paired-sample Wilcoxon Signed Rank tests were used to assess whether the first appearances of quartzite-, shale- and calcareous-endemics within each lineage differed in their timing, and also to evaluate whether the timing of these shifts differed from that of the onset of seasonality. Finally, to evaluate whether the delayed occupation of non-quartzitic substrata was a consequence of the contraction of pre-existing thicket/forest elements rather than recent exposure of these substrata, the contingent states test of Sillén-Tullberg [92] was used to evaluate whether transitions to these substrata was significantly associated with branches optimized as having a seasonally arid precipitation regime. All statistical tests were run in R v2.13.2 [93].

### Diversification rate analyses

Tests for shifts in diversification rate were performed using the delta-AICrc test [94,95]. This method fits rate-constant and rate-variable birth-death or pure-birth models to a given MCC tree, the best-fit model then being selected by evaluating alternative model fits under the Akaike Information Criterion (AIC). Rate-variable models include the exponential and linear density-dependent models, as well as a Yule two-rate model. Log-lineages-through-time (LTT) plots were generated for each clade in order to depict lineage accumulation graphically, and to compare clade age and lineage accumulation rates between groups. LTT plots were calculated using the package ape v3.0–8 [96] in R v2.13.2 [93]. Time- and diversity-dependent models of diversification were fitted using the fitdAICrc function of the package laser v2.4–1 [97] in R v2.13.2 [93].

## Results

### Evaluation of dating analyses

Large effective sample sizes (>200) indicated that run-times of the MCMC chains in all 14 dating analyses were sufficient to ensure effective parameter estimation. Based on the standard deviations of the UCLN relaxed clocks, a strict molecular clock was rejected for all groups, justifying the use of relaxed clock models throughout (Table B in S1 File). Furthermore, the covariance parameter, which serves as a measure of rate autocorrelation, consistently justified our use of the UCLN clock model (Table B in S1 File).

#### Topology

The MCC trees obtained by BEAST for Orchidaceae and Poales were robustly supported and largely congruent with topologies generated by previous analyses (Figures A and B in S1 File). Similarly, the MCC trees for the 12 species-level data sets were robustly supported and generally congruent with previously published phylogenetic reconstructions (Figures C-L in S1 File). Most cases of incongruence between previously published and current tree topologies were minor, being confined to younger nodes which were generally poorly supported in both published and current topologies. One case of significant topological incongruence relates to the position of *Askidiosperma* in the *Elegia-Thamnochortus* clade: where it had previously been reconstructed as sister to *Elegia* [35], our analyses identify it as sister to *Thamnochortus* + *Rhodocoma*.

Matching previously published results, the nuclear and plastid data for *Satyrium* and Danthonioieae reflected disagreement in the placement of a number of taxa (Figures F and G in S1 File). This incongruence was addressed by splitting conflict taxa into their nuclear and plastid counterparts and allowing these to be resolved separately following the approach of Pirie *et al*. [56]. For both *Satyrium* and the two danthonioid clades (*Pentameris* and *Tribolium*), phylogenetic reconstruction then rendered the same tree topology as that resolved by previous reconstructions [55,56]. In the case of *Satyrium*, conflict between nuclear and plastid accessions did not affect any of the Cape taxa and so did not affect any of the downstream analyses done here. By contrast, seven and two Cape taxa were affected by gene incongruence in *Pentameris* and *Tribolium*, respectively, which impacted the outcome of subsequent ancestral state reconstructions.

#### Divergence times

Crown age estimates for the major families included in our higher-level dating analyses (Orchidaceae; Cyperaceae, Restionaceae and Poaceae as part of Poales) ranged from Late Cretaceous to Oligocene (Figures A and B in S1 File), and were variably younger or older than published age estimates (Table D in S1 File). The estimated crown node age for Orchidaceae attained here (75.0 Ma) is broadly consistent with the estimate obtained by Gustafsson *et al*. (77.0 Ma; [70]), the same dataset and dating method (BEAST) being used to derive both. Within Poales, crown node ages for the three major families, Cyperaceae, Restionaceae and Poaceae, were dated to 48.8, 60.1 and 56.2 Ma, respectively. While the date for Cyperaceae matches a recently published molecular estimate (Cyperaceae, ~48 Ma; [71]) and the date for Restionaceae is broadly consistent with the fossil record (Restionaceae, ~64–71 Ma; [79]), that for Poaceae is substantially younger than previously reported estimates. In the species-level analyses, estimated crown ages ranged from Oligocene or Early Miocene (e.g. *Elegia-Thamnochortus*) to Late Miocene (e.g. *Tribolium* and *Stoebe*), with most clades originating in the interval 10–22 Ma.

A comparison of our age estimates for selected nodes, with published estimates, reveals considerable discrepancy (Table D in S1 File). Only in four cases (Orchidaceae, *Elegia-Thamnochortus*, *Moraea*, *Protea*) are published age estimates similar to those obtained here. For the remaining eight comparisons, age discrepancies (the difference between published and those reported here, expressed as a percentage of the former) range from -59.7% to 18.1%. In contrast to the relaxed clock model applied in this study, most published dates were obtained using methods which assume rate autocorrelation (i.e. nonparametric rate smoothing [98], penalized likelihood [99] and the relaxed Bayesian clock implemented in Multidivtime [100,101] and these gave dates that were consistently older (Figure P in S1 File). Where previously published age estimates were obtained using BEAST, however, age estimates were variably older or younger. This suggests a role for methodology in explaining these discrepancies, the mean percentage discrepancy differing significantly between comparisons involving published dates obtained under the assumption of rate autocorrelation (as indicated above) versus those obtained using BEAST (Figure P in S1 File: t = -2.46, df = 11, p = 0.032).

### Historical habitat shifts

**Fig 2:**
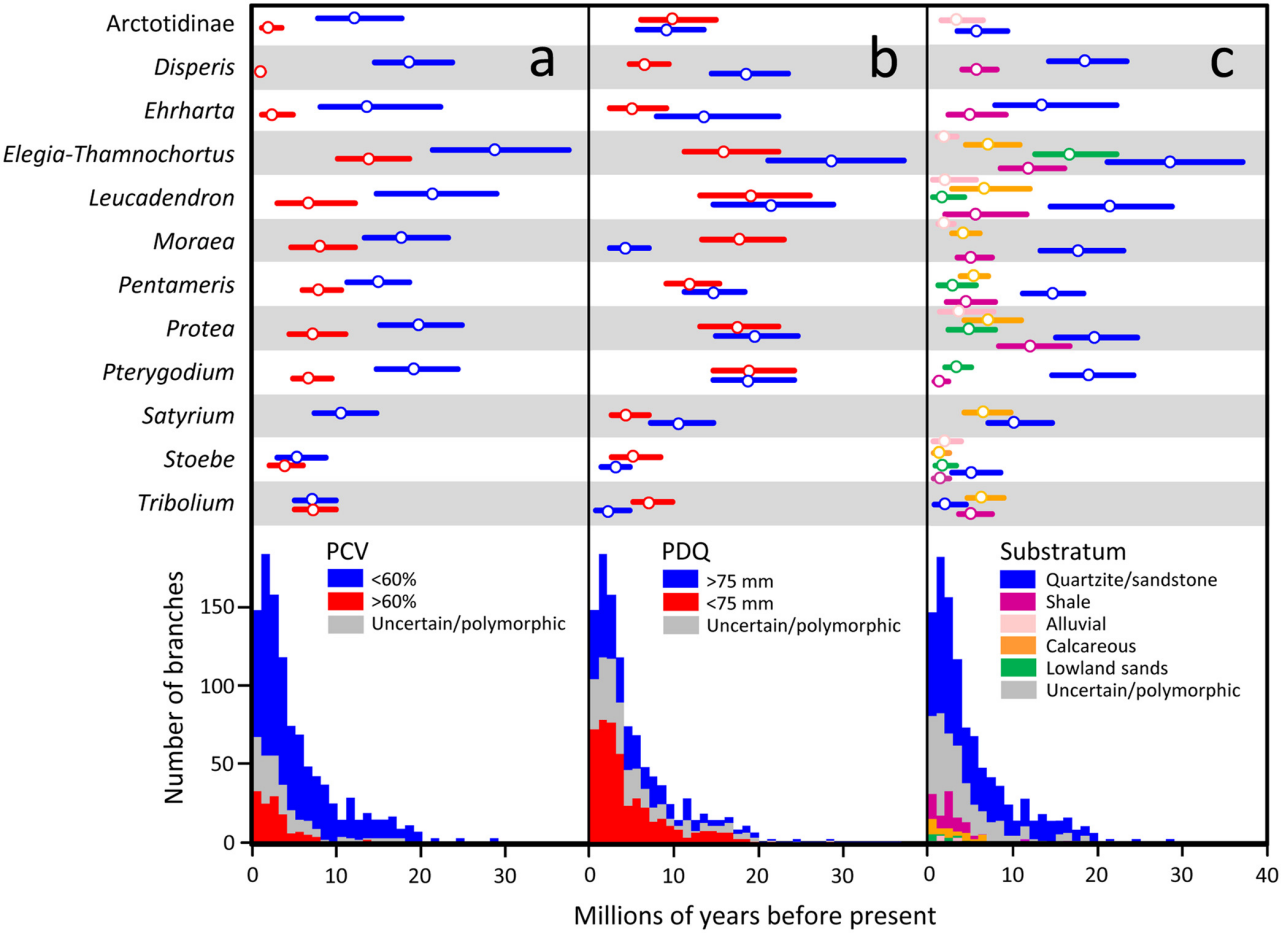
Habitat transitions over time across 12 CFR clades. Upper panel: Error plots (median ± 0.95 HPD) depicting, for each clade, the earliest transition to each alternative habitat state defined under: a) coefficient of variation of monthly precipitation (PCV); b) precipitation during the driest quarter (PDQ); and c) substratum (bedrock geology). Lower panel: Stacked frequency histograms depicting temporal change in the numbers of branches, across all 12 clades, reconstructed as possessing each alternative habitat state.

Figures M-O (in S1 File) depict reconstructions of the three habitat variables. The results of these reconstructions are summarized in Fig 2, which indicates the temporal distribution of branches (across all clades) reconstructed as being associated with each habitat state, as well as the timing of the earliest transitions to each state shown by each lineage (also listed in Table E in S1 File). For *Pentameris* and *Tribolium*, comparison of the timing of habitat shifts in trees with conflict taxa occupying their nuclear- versus their plastid-suggested positions revealed no major differences (Table E in S1 File). Hence, only the reconstructions based on the tree containing the plastid-suggested positions were included in subsequent statistical analyses.

#### Coefficient of variation of monthly precipitation (PCV)

Habitat reconstructions consistently identified associations with aseasonal-rainfall environments (PCV <60%) as ancestral (Figure M in S1 File). Moreover, with the exception of a single divergence event in *Elegia-Thamnochortus* (13.5 [9.6, 18.0] Ma), all divergences prior to 7.6 Ma were associated with aseasonal rainfall conditions, this date marking the start of a sequence of transitions to seasonal environments (PCV >60%; Fig 2a). Across all groups, therefore, the earliest transitions to seasonal rainfall environments were consistently and significantly younger than their earliest occurrences in aseasonal rainfall environments (Fig 2a; paired-sample t test: t = 6.115, df = 10, p < 0.001), having a mean (± s.d.) age of 5.7 ± 3.7 Ma (Table E in S1 File). This value does not differ significantly from the proposed timing of tectonic uplift at the Miocene-Pliocene boundary (5 Ma [21]; one-sample t test: t_5 Ma_ = 0.672, df = 10, p = 0.517), but it does differ from the Late Miocene initiation of Benguela upwelling (10–14 Ma [16,18]; one-sample t test: t_10 Ma_ = -3.848, df = 10, p = 0.003; t_14 Ma_ = -7.464, df = 10, p < 0.001).

#### Precipitation in the driest quarter (PDQ)

The root nodes of seven clades were reconstructed as being associated with environments receiving >75 mm of precipitation in the driest quarter (Figure N in S1 File; *Satyrium*, *Disperis*, *Elegia-Thamnochortus*, *Leucadendron*, *Protea*, *Ehrharta* and *Pentameris*). In Arctotidinae, however, the root node was uncertain, while in *Stoebe*, *Tribolium*, *Moraea* and *Pterygodium* the root node was reconstructed as being associated with periodic or chronic aridity (PDQ <75 mm). Overall, therefore, associations with environments characterized by aridity did not arise significantly earlier or later than those with perennially mesic conditions (Fig 2b; paired-sample t test: t = 0.849, df = 11, p = 0.207). Compared with the earliest transitions to environments characterized by strong rainfall seasonality (PCV >60%), however, the earliest transitions to environments characterized by regular aridity were consistently and significantly older (Fig 2a and 2b; paired-sample Wilcoxon Signed Rank test: V = 55, p = 0.003). Across all groups, the earliest associations with habitats experiencing periodic or chronic drought ranged from 4.1 [2.25, 6.61] Ma (*Satyrium*) to 18.9 [12.7, 25.6] Ma (*Leucadendron*), with a mean (± s.d.) of 11.2 ± 5.8 Ma (Table E in S1 File). This value differs significantly from the from the proposed timing of tectonic uplift at the Miocene-Pliocene boundary (5 Ma [21]; one-sample t test: t_5Ma_ = 3.750, df = 11, p = 0.003), but not from the Late Miocene initiation of Benguela upwelling (10–14 Ma [16,18]; one-sample t test: t_10Ma_ = 0.740, df = 11, p = 0.475; t_14Ma_ = -1.669, df = 11, p = 0.123).

#### Substrate type

Quartzite-endemism (mostly to Table Mountain Group-derived substrata) was reconstructed as the ancestral substratum association in ten of the 12 clades studied, the root nodes of Arctotidinae and *Tribolium* being uncertain with respect to substrate preference (Figure O in S1 File). The earliest shifts to shale substrata (mostly Malmesbury, Bokkeveld and Witteberg Groups) within each group ranged from 1.1 [0.4, 2.1] Ma (*Pterygodium*) to 11.8 [8.0, 16.3] Ma (*Protea*), with the majority of shifts taking place in the interval 4.3 [2.0, 7.6]-6.5 [4.5, 8.9] Ma. Considered across all groups, the earliest transitions to shale substrata were consistently and significantly younger than the earliest instances of quartzite-endemism (Fig 2c; paired-sample-test: t = 5.022, df = 9, p < 0.001), having a mean (± s.d.) of 5.6 ± 3.6 Ma (Table E in S1 File). This value does not differ significantly from the proposed timing of tectonic uplift at the Miocene-Pliocene boundary (5 Ma [21]; one-sample t test: t_5Ma_ = 0.568, df = 9, p = 0.584).

The possibility that transitions onto shale were a consequence of the development of arid or seasonal conditions in shale-based environments is not supported by our data. In the context of the CFR, 84% of the area covered by shale falls within the aseasonal rainfall zone (PCV <60%) and 27% in areas experiencing little aridity at all (PDQ >75 mm; Table 3). Thus, our reconstructions indicate that only five transitions onto shale took place within the context of a seasonal rainfall regime, compared with 36 in an aseasonal context, and this identifies these variables as being evolutionarily unrelated (Sillén-Tullberg test [65,92]: Fisher exact odds ratio = 0.645, P = 0.526). Although transitions onto shale are significantly over-represented on branches associated with PDQ <75 mm (27, compared with 10 transitions on branches with PDQ >75 mm; Fisher exact odds ratio = 2.509, P = 0.012), substantial delays (mean ± s.d. = 6.6 ± 6.0 Ma; N = 10) between the times when study lineages first occupied habitats with PDQ <75 mm and when they first adapted to shale substrata implies that the former did not constrain the latter. These transitions only occurred synchronously in *Ehrharta*.

Seven lineages (*Elegia-Thamnochortus*, *Leucadendron*, *Pentameris*, *Protea*, *Satyrium*, *Stoebe*, and *Tribolium*) showed shifts to calcareous substrata (mostly Bredasdorp Group and Langebaan Formation consolidated aeolianites, calcarenites and calcretes). Except for *Stoebe* that first occupied calcareous substrata about 1.4 [0.7, 2.3] Ma, the earliest transitions onto such substrata all occurred in the interval 4.0 [2.5, 5.8]-7.0 [3.9, 10.7] Ma, with a mean (± s.d.) of 5.4 ± 1.9 Ma across all groups (Table E in S1 File). Across all lineages, associations with calcareous substrata arose significantly earlier than the earliest instances of quartzite-endemism (paired sample t test: t = 3.298, df = 7, p = 0.007). The mean age of the earliest transitions to calcareous substrata, across all groups, did not differ significantly from the proposed timing of tectonic uplift at the Miocene-Pliocene boundary (5 Ma [21]; one-sample t test: t_5Ma_ = 0.642, df = 7, p = 0.542).

With a single exception (*Elegia-Thamnochortus*; 16.6 [12.2, 21.8] Ma), the earliest occupations of the unconsolidated quartzose aeolian sands that cover large parts of the western coastal plain of the CFR (mostly Springfontyn Formation) was recent, ranging from 1.6 [0.2, 4.0] to 4.7 [2.1, 7.6] Ma (Table E in S1 File). Excluding the *Elegia-Thamnochortus* outlier, this yielded a mean (± s.d.) age of 2.8 ± 1.2 Ma, which differs significantly (younger) from the proposed timing of tectonic uplift at the Miocene-Pliocene boundary (5 Ma [21]; one-sample t test: t_5Ma_ = -4.006, df = 4, p = 0.016).

### Patterns of diversification

**Fig 3:**
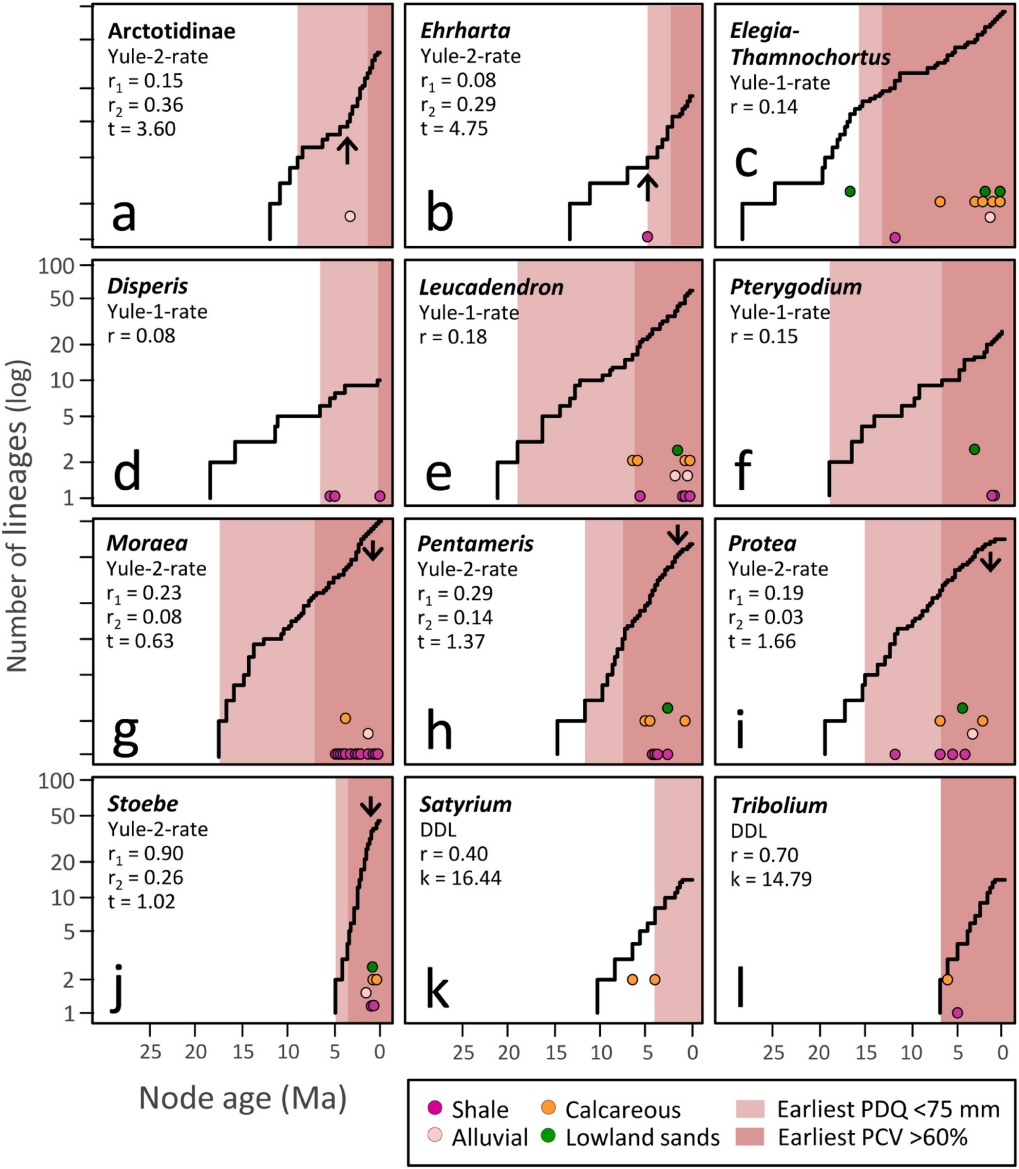
Lineage-through-time plots and best-fit diversification rate models for the 12 CFR clades. Log-lineage-through-time plots for the 12 clades, with the best-fit models of diversification and associated parameter estimates shown. Where the best-fit model invoked two rates, arrows are used to indicate the timing of the rate shift, upward and downward arrows indicating accelerations and decelerations, respectively. For each clade, initial shifts to environments characterized by PDQ <75 mm and PCV >60% are indicated by pale and dark shading, respectively. Shifts to non-quartzitic substrata are indicated by colour-filled circles.

Patterns of lineage accumulation across the twelve lineages are depicted as LTT plots (Fig 3). Lineage accumulation in four of the 12 groups proceeded at more or less constant rates (Fig 3c–3f; *Elegia/Thamnochortus*, *Disperis*, *Leucadendron*, *Pterygodium*), diversification in these lineages being best described by rate-constant, pure-birth models (Fig 3c–3f). Diversification rates fluctuated in the remaining eight lineages but only two, Arctotidinae (Fig 3a) and *Ehrharta* (Fig 3b) showed significant rate increases, at 3.6 and 4.8 Ma respectively. The remainder showed diversification slow-downs, either at a specific point in time (Yule two-rate model; Fig 3g–3j) or else with diversification decelerating as a function of lineage number (DDL model; Fig 3j and 3k).

## Discussion

Our findings confirm the utility of the geoecodynamic approach in testing competing palaeoenvironmental hypotheses. In this study, we show that the genomic record of Cape angiosperm clades carries the signature of Neogene events affecting both the landscape and regional climate of the South African CFR. Habitat reconstructions across the 12 sampled clades exhibit remarkably consistency in the environmental conditions identified as ancestral, and the timings of shifts to alternative environments. The discovery of such consistent patterns across independent lineages suggests that they are a product of broad-scale environmental change. Although climatic and geological changes may have stimulated radiations in some lineages, the signal is by no means ubiquitous.

### Climate and landscape evolution

Habitat reconstruction consistently identified associations with aseasonal-rainfall environments (PCV <60%) as ancestral (Fig 2a; Figure M in S1 File). Moreover, except for a single divergence event in *Elegia-Thamnochortus*, all divergences prior to 7.7 Ma were associated with aseasonal rainfall conditions, this date marking the start of a sequence of transitions to seasonal environments (PCV >60%; Fig 2a). The much earlier transition to seasonal conditions in *Elegia-Thamnochortus* (13.5 [9.6, 18.0] Ma) is plausibly an analytical artefact, possibly reflecting extinction (of aseasonal-rainfall species) along the branch leading to *Dovea macrocarpa* [102]. For six of the 12 lineages studied, the earliest transitions (median estimates) to seasonal environments antedate Pliocene uplift (3–5 Ma) though in only two instances do the 95% confidence intervals exclude a date of 5 Ma (Fig 2a). Thus, while it is impossible to dismiss entirely suggestions linking the present-day seasonality of the western CFR to uplift-mediated formation of a summer rain shadow [21,103], an earlier onset of seasonality at ~6.5–8 Ma appears more likely. This result is consistent with the first appearance of winter-rain adapted Aizoaceae pollen at ODP site 1085 ~8 Ma [8], and with isotopic signatures from ratite eggshells, which date the initial differentiation of distinct winter- and summer-precipitation regimes, in southern and central Namibia respectively, to ~7 Ma [27]. Though some uncertainty persists about the precise mechanisms involved [21], the development of summer aridity along the west coast of southern Africa is commonly linked to progressive strengthening of Benguela upwelling [16,104] after its initial development 10–14 Ma [17,18]. The onset of summer aridity—the winter rainfall regime—is clearly not attributable to a dramatic drop in sea surface temperatures taking place ~2.8 Ma [19,20].

While the seasonally arid rainfall regime of the western CFR dates to the Late Miocene, climates characterized by regular aridity (PDQ <75 mm) appear to have originated earlier, probably in the early Middle Miocene (Fig 2b; Figure N in S1 File). Paired-sample Wilcoxon Signed Rank tests identify shifts to PDQ <75 mm as being consistently and significantly younger than shifts to PCV >60%, confirming that the initial development of aridity in the CFR was not linked to the onset of strong rainfall seasonality. Instead, the initial appearance of aridity appears to have been associated with the formation of somewhat aseasonal conditions, more closely resembling the contemporary climates of the Agulhas Plain and the Little Karoo. Supporting this idea, Roberts and Brink [105] interpret the presence of giant Ostrich-like birds and a giant form of the snail *Trigonephrus* cf. *globulus* in reportedly 10–12 Myr-old calcareous aeolianites from the Cape west coast (Prospect Hill Formation; but see [106] who date the oldest of these sediments to 5.2–5.9 Ma) as indicative of a dry climate in which rainfall was more seasonally consistent than it is at present. For the 12 study lineages, the earliest transitions to habitats with PDQ <75 mm span the period 4.1–18.9 [2.3, 23.9] Ma, with five of these shifts taking place before 10 Ma (Fig 2b). Based on these dates, we infer that the initial development of Miocene aridity in the CFR was likely prompted by Antarctic ice sheet expansion following the Mid-Miocene Climatic Optimum [10], this event also being implicated in the hyper-aridification of the Namib and Kalahari deserts, also initiated at ~13–17 Ma [103,107–109].

Consistent with recent cosmogenic evidence testifying to the protracted stability of the CFR mountain uplands, at least since the Pliocene and possibly earlier [31], our substratum reconstructions consistently identify associations with the quartzitic substrata (mostly Table Mountain Group-derived) of the Cape Fold Belt as ancestral and ancient, in two instances possibly being of Late Paleogene age (Fig 2c; Figure O in S1 File). However, our reconstructions also support recent re-interpretation [33] of the extensive, surficial exposure of clay-rich, shale and mudstone substrata (mostly Malmesbury, Bokkeveld and Witteberg Groups) at low elevations as a product of renewed erosion associated with Early Pliocene tectonism. Following an extended period (Late Oligocene-Pliocene) during which movement onto such substrata was limited, being apparent in only two clades (*Elegia-Thamnochortus*, 11.7 [8.3, 15.8] Ma; *Protea*, 11.8 [8.0, 16.3] Ma), we observe a flurry of such transitions in the interval 4.3 [2.0, 7.6]-6.5 [4.5, 8.9] Ma. This result is entirely consistent with the hypothesis [33] that exposure of shale and mudstone substrata was limited for much of the Miocene, after which it was greatly enhanced by renewed erosion resulting from the Post African II uplift event (~5 Ma [103]). The general youthfulness of succulent Karoo- [110] and renosterveld-centred plant lineages [52,53] provides further support, both of these vegetation types associating primarily with shale substrata in the CFR. A prime example is Heliophileae which, with its estimated crown age of 3.7–5.4 Ma [111], represents one of the youngest radiations documented for the Cape flora.

While delayed exposure of shale substrata provides one explanation for the generally late appearance of shale-endemic lineages, an alternative is that these substrates were exposed much earlier, but that the delayed establishment of aridity/seasonal-aridity was required to facilitate their occupation by the lineages surveyed here. The onset of aridity is commonly believed to have precipitated the decline of thicket/forest elements, which may previously have dominated the CFR shales, so facilitating the occupation of the latter by drought-adapted, scrub lineages [14,48,49]. Our data do not, however, support this alternative explanation of a climatic trigger. Firstly, in the context of the CFR, 84% of the area covered by shale falls within the aseasonal rainfall zone (PCV <60%) and 27% in areas experiencing little aridity at all (PDQ >75 mm). Secondly, tests of evolutionary association indicate that transitions onto shale have not generally taken place in the context of a seasonal rainfall regime (Sillén-Tullberg test; [92]). Finally, although movements onto shale are significantly over-represented on branches associated with PDQ <75 mm, substantial delays (mean ± s.d. = 6.6 ± 4.9 Ma; N = 10) between the times of first occupancy of habitats with PDQ <75 mm and those with shale substrata, implies that transitions to the former did not constrain occupation of the shale substrate.

Marked synchronicity of the earliest transitions to calcareous substrata (mostly Bredasdorp Group and Langebaan Formation consolidated aeolianites, calcarenites and calcretes) is associated with the CFR coastline, and the combined signal in the sampled clades provide strong evidence for the availability of such substrates from around the Miocene-Pliocene boundary. Except for *Stoebe* which occupied calcareous substrata only recently, about 1.4 [0.7, 2.3] Ma, the earliest occupations of these substrata all occurred between 4.0 [2.5, 5.8] and 7.0 [3.9, 10.7] Ma. Although the CFR coastal platform likely has a long history of marine sediment deposition, possibly spanning the entire Cenozoic [105], the extent of these deposits as well as their aerial exposure has likely varied through time, because of sea level changes caused by global temperature fluctuations and tectonic uplift of the coastal margin [105]. The apparently coordinated occupation of calcareous substrata reported in this study coincides roughly with the end of a major sea level transgression at the Miocene-Pliocene boundary, with its high stand variously dated to 4.8–5.2 Ma [26,112,113], but predates a more recent high stand proposed at 3.2–2.9 Ma [114]. The implication is that the Early (but not the Late) Pliocene transgression was of sufficient magnitude to precipitate a major episode of biotic turnover along the coastal margin. The dramatic scale of the earlier transgression is corroborated by the presence of associated marine deposits at present-day elevations up to 330 m a.s.l., although the modern positions of some of these landforms are probably partly attributable to post-African II uplift [22].

With a single exception (*Elegia-Thamnochortus*; 16.6 [12.2, 21.8] Ma), the earliest occupations of the unconsolidated quartzose aeolian sands, covering large parts of the western coastal plain of the CFR (mostly Springfontyn Formation), was recent (1.6 [0.2, 4.0]-4.7 [2.1, 7.6] Ma). While the errors in molecular dates of most of these shifts preclude a Middle Pleistocene origin for these sediments, as suggested by [115], an Early Pleistocene origin is possible. Based on the general consistency of this pattern, we interpret the very early shift to these lowland sands shown by *Elegia-Thamnochortus* as an analytical artefact, possibly reflecting extinctions along the branch subtending *Elegia acockii* (0–16.6 Ma).

In summary, the overall picture to emerge from our reconstructions is that Early Miocene environments of the CFR were generally wetter than at present, with quartzitic substrata dominating the land surfaces. Although our data provide no support for the widespread occurrence of silcrete substrata at lower elevations at this time [33], this finding is inevitable given that these substrates have been largely stripped away. The Middle Miocene (13–17 Ma) saw the development of perennial to weakly-seasonal aridity, with the more strongly seasonal rainfall regime of the west coast arising later, probably ~6.5–8 Ma. Although the Late Miocene may have seen some exposure of the underlying shale substrata, the present-day substrate diversity of the CFR appears to be a product largely of Pliocene-Pleistocene events, especially renewed erosion following the post-African II uplift, and the reworking of sediments across the coastal platform as a result of temperature-related sea level fluctuations and tectonic uplift.

### Floristic diversification

Although both the appearance of seasonal aridity ~8 Ma [14,46,50] and the exposure of novel substrata from ~5 Ma onwards [33] have been proposed as potential triggers of floristic radiation in the CFR, our analyses do not reveal consistent pulses of accelerated diversification which are readily attributable to these events. Diversification in four lineages is best described by rate-constant, pure-birth models (Fig 3c–3f), while a further six lineages show diversification slow-downs (Fig 3g–3l). Thus, of all the clades studied, only Arctotidinae (Fig 3a) and *Ehrharta* (Fig 3b) show significant diversification rate increases (at 3.6 and 4.8 Ma respectively) and only in the latter does this coincide with a transition to environments characterized by aridity and/or non-quartzitic soils. It is not possible, however, to discount a role for aridification and substrate change in stimulating accelerated speciation in *Tribolium* (Fig 3l) or *Stoebe* (Fig 3j), both of which are too young (~7.5 and 5.0 Ma, respectively) to allow for the discovery of significant rate shifts in response to events ~5–8 Ma. In addition, although our phylogenetic trees are generally well sampled, we cannot discount entirely the effects of incomplete taxonomic sampling, and the general failure of taxonomists to recognize cryptic diversity, which in turn underestimates signatures of accelerated speciation [116], particularly in the recent past. Notwithstanding, the overall pattern to emerge from our data is that, while environmental change in the late Neogene may have stimulated adaptive radiation in some lineages, this effect is far from ubiquitous. Indeed, the signatures of diversification within each of the 12 sampled clades reveal individualistic evolutionary responses.

## Conclusions

The most striking feature of our results is the remarkable consilience, across lineages sampled, with regard to the types of habitat identified as ancestral, and the sequence and timing of occupation of geologically and climatically younger conditions. Such congruence is an unlikely outcome of chance, and these patterns are best interpreted as a broad biotic response to climatic and geomorphological events shaping Neogene palaeoenvironments across the CFR. The strength and consistency of these signals serve to highlight the power of the geoecodynamic approach in resolving palaeoenvironmental events, particularly where the geochronological resolution provided by direct geological proxies is limited. For the CFR, insights gained from cosmogenic isotopes have to date been limited to the Late Neogene, while thermochronological profiles lack the spatial precision required to resolve and distinguish events that have reworked or shaped landforms within continental surfaces [31,37,117]. In contrast, the paleoenvironmental signals recorded in the extant Cape flora resolve specific habitat associations since the Early Neogene.

The accuracy of geoecodynamic interpretation depends, of course, on the validity of the numerous assumptions underpinning the inference of molecular dates [118] and ancestral habitat types [119]. While the robustness of our conclusions to alternative assumptions warrants evaluation, the large scale of our study renders this unfeasible. We have, however, taken care to follow analytical protocols which make sense given the available data and, crucially, which conform to accepted best-practice. Our use of lognormal priors to specify fossil-calibrations, as well as their application to stem rather than crown nodes, for example, conforms to recommendations developed in the literature [61,62,63]. Nonetheless, we readily acknowledge that the form of priors on fossil-based calibrations remains an important and contentious issue (e.g. [120]) and that our results warrant careful interpretation.

While lineage selection also has the potential to introduce biases, this is probably not a significant problem in this study. Our lineage sample is phylogenetically disparate and includes clades whose habitat associations collectively represent a diversity of environments found in the CFR. Although several of the lineages sampled associate predominantly with quartzitic fynbos vegetation (e.g. *Elegia-Thamnochortus*, *Protea*, *Leucadendron*, *Stoebe*), the other clades are most prominent and diverse in non-fynbos vegetation (e.g. Arctotidinae, *Disperis*, *Ehrharta*, *Pterygodium*, *Satyrium*, *Tribolium*), with two being rather catholic in terms of habitat preference (e.g. *Moraea*, *Pentameris*).

Historical inference is subject to error from a variety of sources, and geoecodynamics is no exception. With careful application, however, this approach has the potential to yield novel insights which can be integrated with complementary insights gained from other lines of evidence, to obtain consilience and thus gain a broader and better-resolved picture of landscape history.

## Supporting Information

S1 File Zip file containing Supporting Information Figures A-P and Tables A-F. (ZIP)
