# Supplementary material for: Dated Plant Phylogenies Resolve Neogene Climate and Landscape Evolution in the Cape Floristic Region
Source: PLoS One. 2015 Sep 30;10(9):e0137847. doi: 10.1371/journal.pone.0137847 (PMC4589284; doi:10.1371/journal.pone.0137847)
Supplement: S1 File — (ZIP) [file pone.0137847.s001.zip › Supporting Information 1_S1/Fig A.pdf]

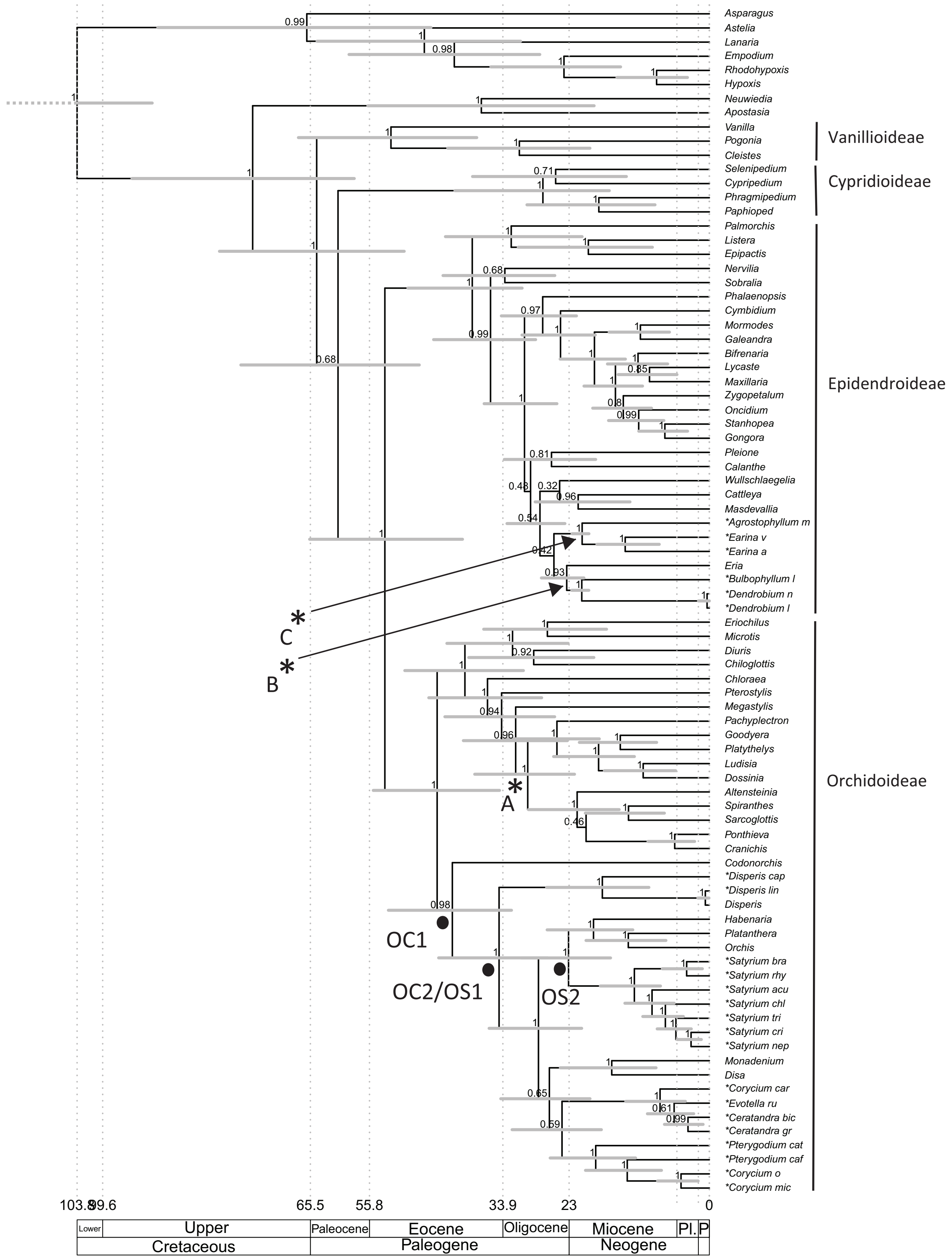

Fig A. Dated Bayesian maximum clade credibility tree for Orchidaceae. Values on nodes are median node ages with bars indicating the 95% credibility intervals (95% HPDs). Calibration nodes are indicated by capital letters A-C (marked with stars; see Table 2 for calibration details). OC1, OC2, OS1 and OS2 depict nodes used as secondary calibration points for species-level dating analyses (filled circles: OC, Coryciinae; OS, *Satyrium*). Taxa added to the baseline data set in order to obtain secondary calibration dates for species-level phylogenies are marked with asterisks (*).
