# Supplementary material for: Dated Plant Phylogenies Resolve Neogene Climate and Landscape Evolution in the Cape Floristic Region
Source: PLoS One. 2015 Sep 30;10(9):e0137847. doi: 10.1371/journal.pone.0137847 (PMC4589284; doi:10.1371/journal.pone.0137847)
Supplement: S1 File — (ZIP) [file pone.0137847.s001.zip › Supporting Information 1_S1/Fig G.pdf]

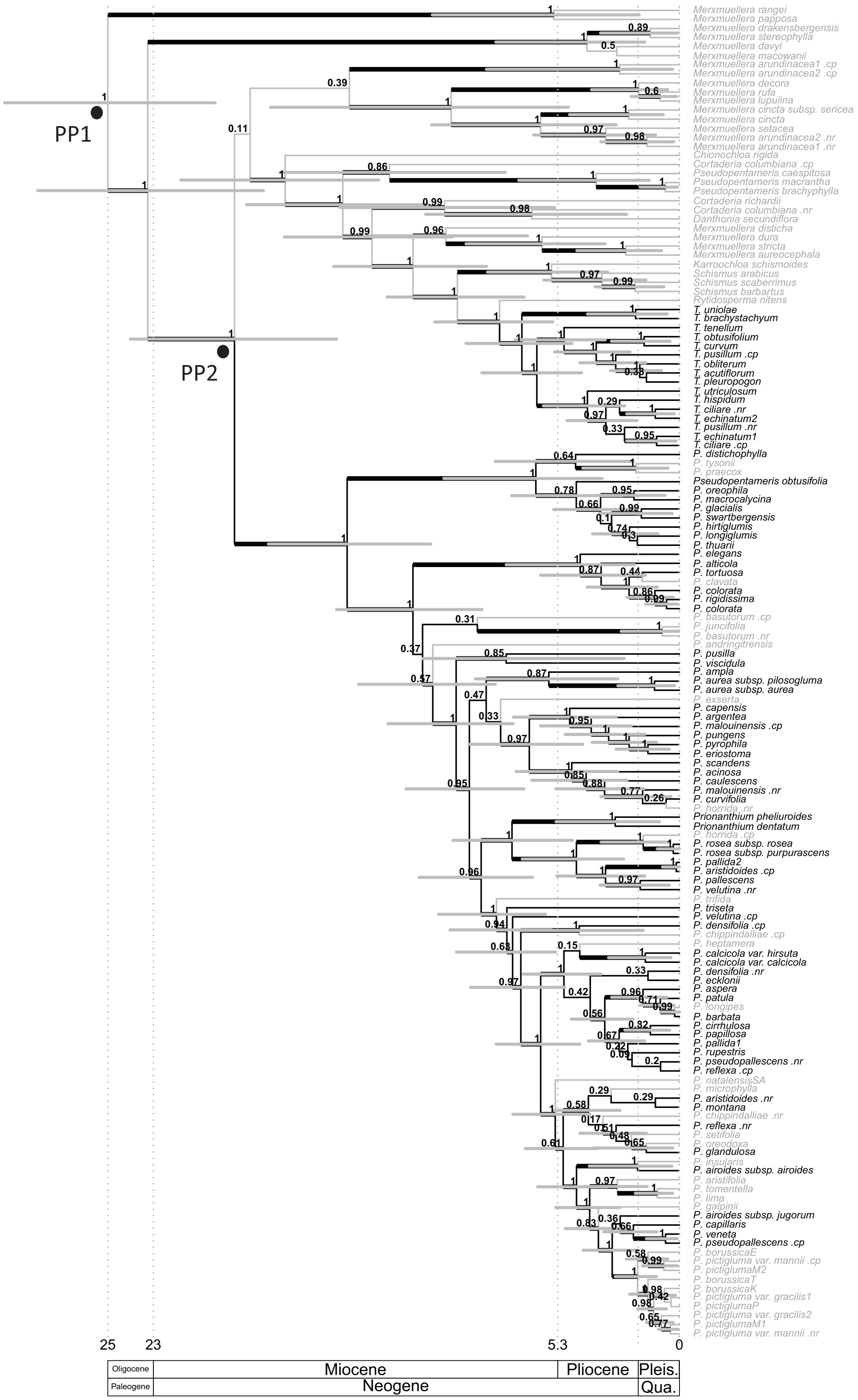

Fig G. Dated Bayesian maximum clade credibility tree for Danthonioideae (*Pentameris* and *Tribolium*). Values on nodes are posterior probabilities. Calibration nodes are indicated by PP1 and PP2 as in the higher-level phylogeny (Poales) from which the calibration was obtained (see Table B in S1 File for calibration details). Taxa in grey font are those for which no georeferenced data were available or which do not occur in the Cape Floristic Region. Nuclear and plastid accessions are suffixed by '.nr' and '.cp', respectively.
