# Supplementary material for: Dated Plant Phylogenies Resolve Neogene Climate and Landscape Evolution in the Cape Floristic Region
Source: PLoS One. 2015 Sep 30;10(9):e0137847. doi: 10.1371/journal.pone.0137847 (PMC4589284; doi:10.1371/journal.pone.0137847)
Supplement: S1 File — (ZIP) [file pone.0137847.s001.zip › Supporting Information 1_S1/Fig H.pdf]

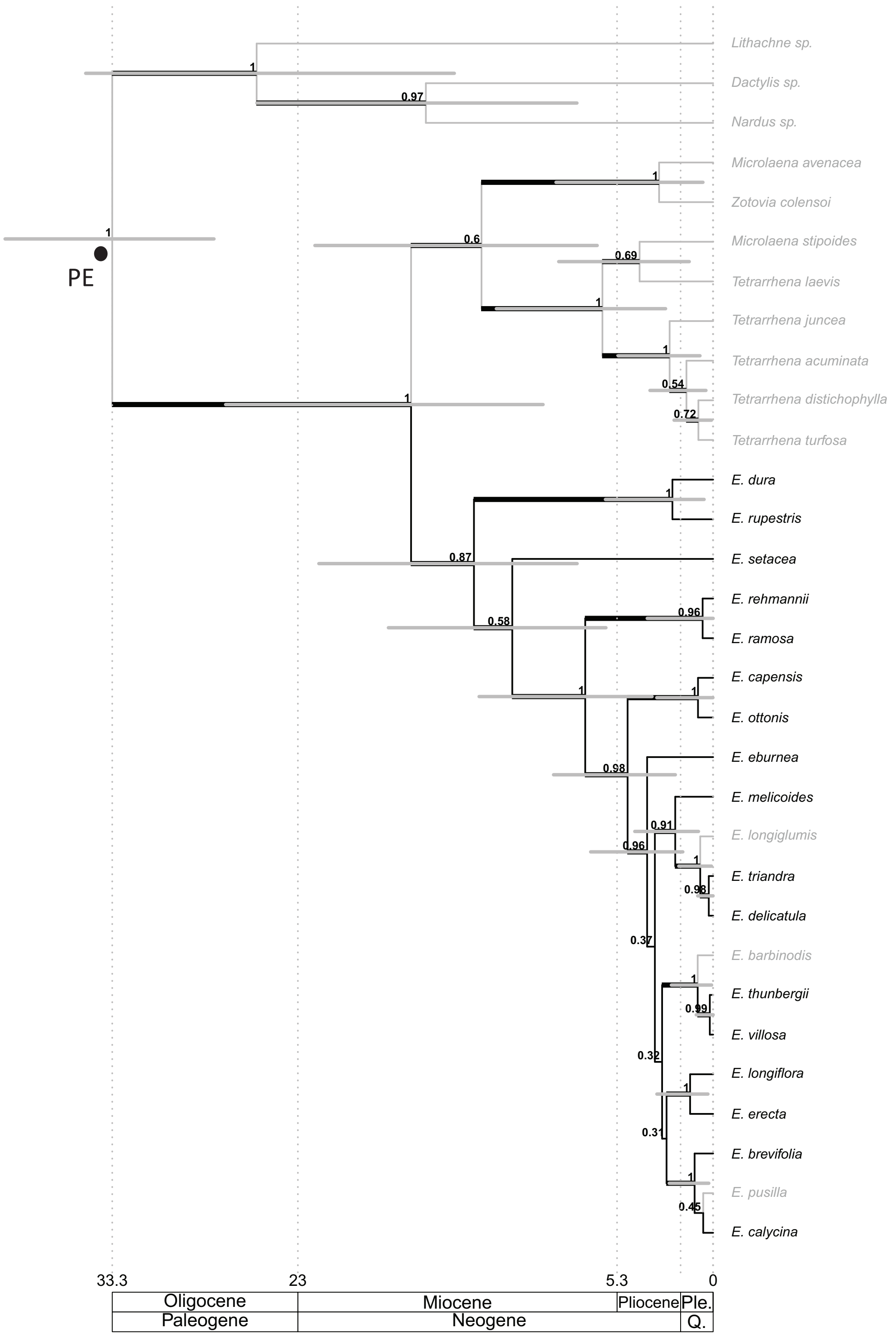

Fig H. Dated Bayesian maximum clade credibility tree for *Ehrharta*. Values on nodes are posterior probabilities. The calibration node is indicated by PE as in the higher-level phylogeny (Poales) from which the calibration was obtained (see Table B in S1 File for calibration details). Taxa in grey font are those for which no georeferenced data were available or which do not occur in the Cape Floristic Region.
