# Supplementary material for: Dated Plant Phylogenies Resolve Neogene Climate and Landscape Evolution in the Cape Floristic Region
Source: PLoS One. 2015 Sep 30;10(9):e0137847. doi: 10.1371/journal.pone.0137847 (PMC4589284; doi:10.1371/journal.pone.0137847)
Supplement: S1 File — (ZIP) [file pone.0137847.s001.zip › Supporting Information 1_S1/Fig O.pdf]

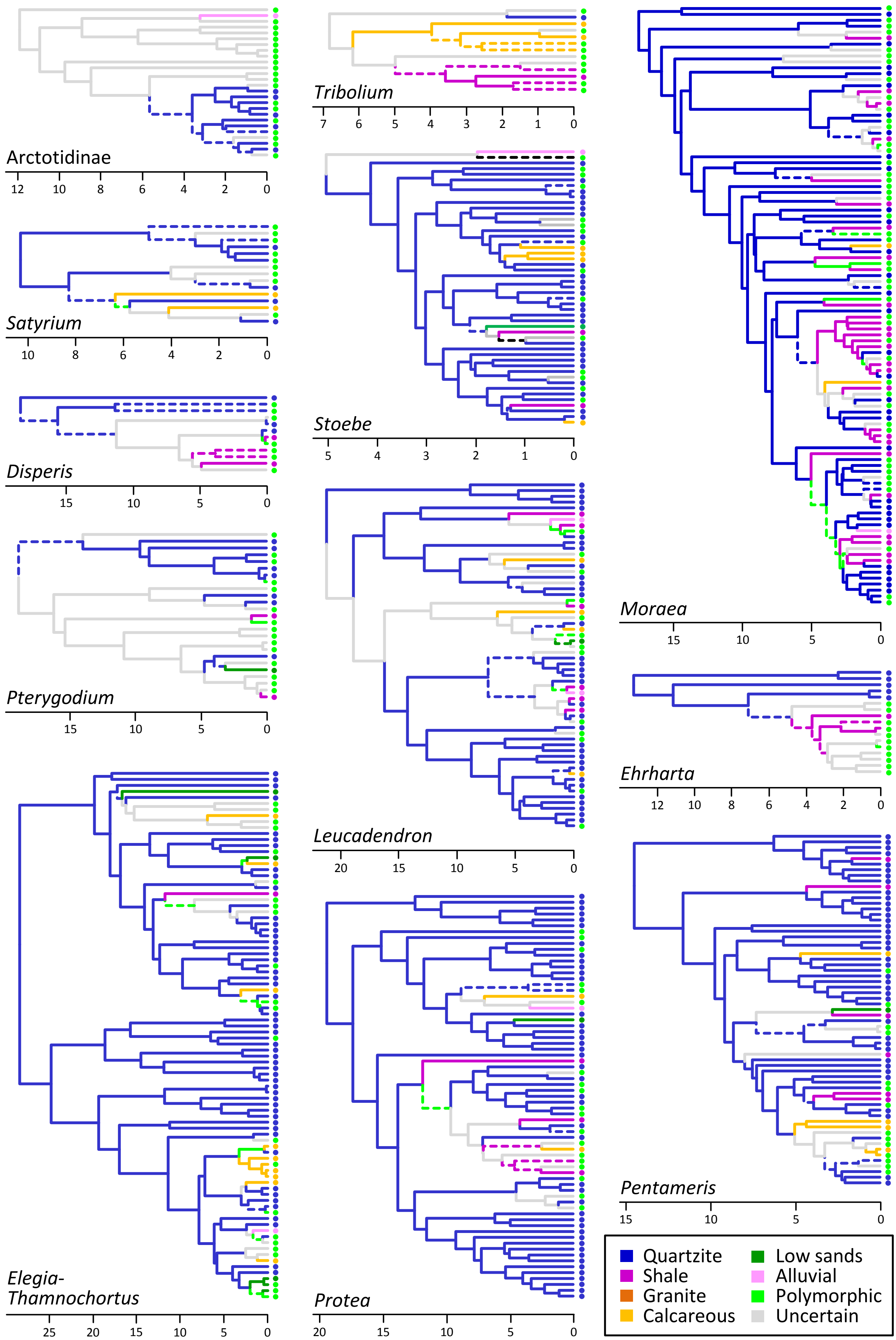

Arctotidinae
12 10 8 6 4 2 0
Satyrium
10 8 6 4 2 0
Disperis
15 10 5 0
Pterygodium
15 10 5 0
Elegia-
Thamnochortus
25 20 15 10 5 0
Tribolium
7 6 5 4 3 2 1 0
Stoebe
5 4 3 2 1 0
Leucadendron
20 15 10 5 0
Protea
20 15 10 5 0
Moraea
15 10 5 0
Ehrharta
12 10 8 6 4 2 0
Pentameris
15 10 5 0
Quartzite
Shale
Granite
Calcareous
Low sands
Alluvial
Polymorphic
Uncertain

Fig O. Lagrange optimization of substratum type on the BEAST maximum clade credibility trees obtained for the 12 study lineages. Shading of filled circles at the branch tips indicate the terminal states, while branch shading indicates reconstructed ancestral states. Solid lines indicate reconstructions having a probability ≥70%, while dashed lines indicate reconstructions having probabilities in the range 55%-70%. Branches attaining a probability <55% for all possible states are marked as uncertain.
