# Supplementary material for: Dated Plant Phylogenies Resolve Neogene Climate and Landscape Evolution in the Cape Floristic Region
Source: PLoS One. 2015 Sep 30;10(9):e0137847. doi: 10.1371/journal.pone.0137847 (PMC4589284; doi:10.1371/journal.pone.0137847)
Supplement: S1 File — (ZIP) [file pone.0137847.s001.zip › Supporting Information 1_S1/Fig P.pdf]

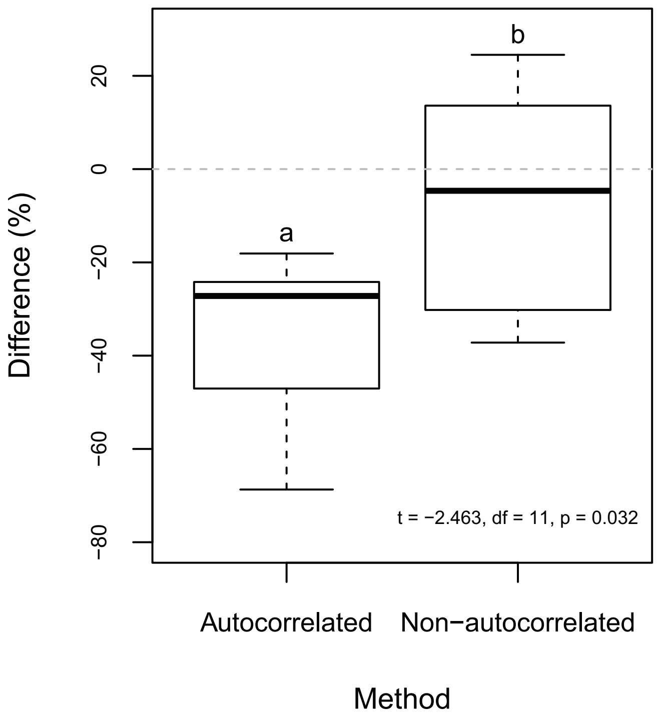

Fig P. Percentage discrepancies between age estimates obtained in this study and those obtained previously using methods which assume rate and those which do not. Discrepancy was determined as the difference between previously published and new age estimates, expressed as a percentage of the former. The mean age discrepancies with published dates based on methods which assume rate autocorrelation and those which do not were significantly different ($t = -2.463$, $df = 11$, $p = 0.032$). Age estimates obtained using methods which assume rate autocorrelation were consistently older.
