# Supplementary material for: Dated Plant Phylogenies Resolve Neogene Climate and Landscape Evolution in the Cape Floristic Region
Source: PLoS One. 2015 Sep 30;10(9):e0137847. doi: 10.1371/journal.pone.0137847 (PMC4589284; doi:10.1371/journal.pone.0137847)
Supplement: S1 File — (ZIP) [file pone.0137847.s001.zip › Supporting Information 1_S1/Supporting Information Reference List.pdf]

Supporting Information References

1. Hoffmann V. Plants as biotic indicators of Neogene palaeoenvironmental evolution in the Cape Floristic Region [MSc]. Cape Town, South Africa: University of Cape Town; 2012.
2. Sauquet H, Weston PH, Barker NP, Anderson CL, Cantrill DJ, Savolainen V. Using fossils and molecular data to reveal the origins of the Cape proteas (subfamily Proteoideae). Mol Phylogen Evol. 2009;51(1):31-43.
3. Chen S, Kim D-K, Chase MW, Kim J-H. Networks in a large-scale phylogenetic analysis: reconstructing evolutionary history of Asparagales (Lilianae) based on four plastid genes. PloS one. 2013;8(3):e59472.
4. Rabosky DL. Likelihood methods for detecting temporal shifts in diversification rates. Evolution. 2006;60(6):1152-64.
5. Ramírez SR, Gravendeel B, Singer RB, Marshall CR, Pierce NE. Dating the origin of the Orchidaceae from a fossil orchid with its pollinator. Nature. 2007;448(7157):1042-5.
6. Gustafsson ALS, Verola CF, Antonelli A. Reassessing the temporal evolution of orchids with new fossils and a Bayesian relaxed clock, with implications for the diversification of the rare South American genus *Hoffmannseggella* (Orchidaceae: Epidendroideae). BMC Evol Biol. 2010;10(1):177.
7. Janssen T, Bremer K. The age of major monocot groups inferred from 800+ rbcL sequences. Bot J Linn Soc. 2004;146(4):385-98.
8. Vicentini A, Barber JC, Aliscioni SS, Giussani LM, Kellogg EA. The age of the grasses and clusters of origins of C4 photosynthesis. Global Change Biol. 2008;14(12):2963-77.
9. Christin P-A, Salamin N, Muasya AM, Roalson EH, Russier F, Besnard G. Evolutionary switch and genetic convergence on rbcL following the evolution of C_4 photosynthesis. Mol Biol Evol. 2008;25(11):2361-8.
10. Bouchenak-Khelladi Y, Verboom GA, Savolainen V, Hodkinson TR. Biogeography of the grasses (Poaceae): a phylogenetic approach to reveal evolutionary history in geographical space and geological time. Bot J Linn Soc. 2010;162(4):543-57.
11. McKenzie RJ, Barker NP. Radiation of southern African daisies: biogeographic inferences for subtribe Arctotidinae (Asteraceae, Arctotideae). Mol Phylogen Evol. 2008;49(1):1-16.
12. Bergh NG, Peter Linder H. Cape diversification and repeated out-of-southern-Africa dispersal in paper daisies (Asteraceae–Gnaphalieae). Mol Phylogen Evol. 2009;51(1):5-18.
13. Verboom GA, Linder HP, Stock WD. Phylogenetics of the grass genus *Ehrharta*: Evidence for radiation in the summer-arid zone of the South African Cape. Evolution. 2003;57(5):1008-21.
14. Linder HP, Hardy CR, Rutschmann F. Taxon sampling effects in molecular clock dating: an example from the African Restionaceae. Mol Phylogen Evol. 2005;35(3):569-82.
15. Schnitzler J, Barraclough TG, Boatwright JS, Goldblatt P, Manning JC, Powell MP, et al. Causes of plant diversification in the Cape biodiversity hotspot of South Africa. Syst Biol. 2011;60(3):343-57.
16. Valente LM, Reeves G, Schnitzler J, Mason IP, Fay MF, Rebelo TG, et al. Diversification of the African genus *Protea* (Proteaceae) in the Cape biodiversity hotspot and beyond: equal rates in different biomes. Evolution. 2010;64(3):745-60.
