# Supplementary material for: Dated Plant Phylogenies Resolve Neogene Climate and Landscape Evolution in the Cape Floristic Region
Source: PLoS One. 2015 Sep 30;10(9):e0137847. doi: 10.1371/journal.pone.0137847 (PMC4589284; doi:10.1371/journal.pone.0137847)
Supplement: S1 File — (ZIP) [file pone.0137847.s001.zip › Supporting Information 1_S1/Table A.docx]

**Table A. List of additional taxa used in higher-level dating analyses, Genbank accession numbers, and specimen vouchers.**

| Family | Taxon | Gene |  |  | Specimen Voucher |
| --- | --- | --- | --- | --- | --- |
|  |  | ndhF | rbcL | matK |  |
| Cyperaceae | *Tetraria involucrata* | - | AM234973 | - | Balele K 33 (NBG) |
|  | *Tricostularia pauciflora* | - | AY725954 | - |  |
| Restionaceae | *Thamnochortus levynsiae* | - | AY690759 | - | Linder HP, Hardy CR, Moline P 7345 |
|  | *Thamnochortus sporadicus* | - | AY690780 | - | Linder HP, Hardy CR, Moline P 7341 |
|  | *Thamnochortus glaber* | - | AY690756 | - | Cowling sn |
|  | *Rhodocoma fruticosa* | - | AY690749 | - | Linder HP, Hardy CR, Moline P 7609 |
|  | *Rhodocoma capensis* | - | AY690748 | - | Linder HP, Hardy CR, Moline P 7248 |
|  | *Askidiosperma paniculatum* | - | AY881410 | - | Linder HP, Hardy CR, Moline P 7378 |
|  | *Chondropetalum nudum* | - | AY881421 | - | Linder HP, Hardy CR, Moline P 7219 |
|  | *Staberoha multispicula* | - | AY881473 | - | Linder HP, Hardy CR, Moline P 7318 |
| Poaceae | *Pentameris aurea* | EU400811 | EU400671 | - | Galley C 47 (Z) |
|  | *Pentameris airoides* | EU400810 | EU400670 | - | Galley C 81 (Z) |
|  | *Tribolium brachystachyum* | EU400823 | EU400677 | - | Verboom GA 593 (BOL) |
|  | *Pseudopentameris macrantha* | EU400816 | DQ887122 | - | Linder HP5470 (BOL) |
| Orchidaceae | *Corycium orobanchoides* | - | - | EU301528 | Pauw A 47 (BOL) |
|  | *Corycium microglossum* | - | - | EU301526 | Pauw A 16 (BOL) |
|  | *Corycium carnosum* | - | AY381115 | EU301524 | Chase MW O-692 (K)/ Pauw A 30 (BOL) |
|  | *Pterygodium catholicum* | - | AY368346 | EU301533 | Chase MW O-1130/ Pauw A 28 (BOL) |
|  | *Pterygodium caffrum* | - | - | EU301525 | Pauw A 27 (BOL) |
|  | *Ceratandra grandiflora* | - | - | EU687535 | Pauw & Liltveld 49 (BOL) |
|  | *Ceratandra bicolor* | - | - | EU301541 | Pauw A 2 (BOL) |
|  | *Evotella rubiginosa* | - | - | EU301508 | Pauw A 4 (BOL) |
|  | *Disperis capensis* | - | AY381120 | AJ310022 | Chase MW O-1203 (K)/MWC 1203 |
|  | *Disperis lindleyana* | - | AY370651 | AY370652 | Chase MW O-696 |
|  | *Earina autumnalis* | - | AF074155 | AF263656 |  |
|  | *Earina valida* | - | AF518051 | AY121741 | C296 |
|  | *Agrostophyllum majus* | - | AF518054 | AY368391 | MWC1402/Chase MW O-562 |
|  | *Bulbophyllum lobbii* | - | AF074115 | AY368395 | Chase MW O-474 |
|  | *Dendrobium lindleyi* | - | GQ248589 | GQ248117 | USBG 99-2351 |
|  | *Dendrobium nobile* | - | FJ216583 | FJ216672 | SMJC-SH |
|  | *Satyrium bracteatum* | - | - | EF612540 | BB2110 (BR, K, NBG, NY) |
|  | *Satyrium rhynchatum* | - | - | EF612588 | BB2155 (BR, NBG, Z) |
|  | *Satyrium acuminatum* | - | - | EF612534 | T18b (Z) |
|  | *Satyrium chlorochorys* | - | - | EF612547 | HK1969 (MAL, PRE,SRGH, UZL) |
|  | *Satyrium trinerve* | - | - | EF612595 | BB2255 (BR, NBG) |
|  | *Satyrium cristatum* | - | - | EF612552 | BB2297 (GRA, NBG) |
|  | *Satyrium nepalense* | - | - | EF612575 | Chase O-539 (K) |
