# Supplementary material for: Dated Plant Phylogenies Resolve Neogene Climate and Landscape Evolution in the Cape Floristic Region
Source: PLoS One. 2015 Sep 30;10(9):e0137847. doi: 10.1371/journal.pone.0137847 (PMC4589284; doi:10.1371/journal.pone.0137847)
Supplement: S1 File — (ZIP) [file pone.0137847.s001.zip › Supporting Information 1_S1/Table B.docx]

**Table B.** **Secondary calibration of species-level dating analyses using posterior age distributions obtained from higher-level dating analyses.**

| Higher-level | Species-level | Node label | Description | Posterior | (Higher level) | Prior (Species level) | | | |
| --- | --- | --- | --- | --- | --- | --- | --- | --- | --- |
|  |  |  |  | Median | 95% HPD | | Mean | SD | 95% CI |
| Ast | Arctotidinae | AA | Crown Cichorioideae | 31.46 | [23.32, 38.90] | | 31.43 | 3.9 | [23.36, 38.64] |
| Ast | *Stoebe* | AS | Crown Gnaphalieae | 18.99 | [11.56, 25.78] | | 19.02 | 3.0 | [13.12, 24.88] |
| Asp | *Moraea* | AM1 | Crown Iridoideae | 34.00 | [26.00, 42.00] | | 34.00 | 4.1 | [26.00, 42.00] |
|  |  | AM2 | Split *Dietes-Ferraria*+*Moraea* | 19.00 | [10.00, 28.50] | | 19.00 | 4.6 | [10.00, 28.00] |
| Orc | Coryciinae (*Pterygodium* and *Disperis*) | OC1 | Split *Codonorchis*-Coryciinae+Orchideae+Disinae | 42.19 | [32.48, 52.70] | | 42.41 | 5.1 | [32.41, 52.41] |
|  |  | OC2 | Split *Disperis-Pterygodium* | 34.53 | [26.53, 44.45] | | 34.92 | 4.4 | [26.29, 43.54] |
| Orc | *Satyrium* | OS1 | Split *Disperis-Satyrium* | 34.53 | [26.53, 44.45] | | 34.92 | 4.4 | [26.29, 43.54] |
|  |  | OS2 | Split Orchideae-*Satyrium* | 23.12 | [16.22, 30.82] | | 23.45 | 3.5 | [16.59, 30.31] |
| Poa | Danthonioideae (*Pentameris* and *Tribolium*) | PP1 | Split Chloridoideae-Danthonioideae | 23.73 | [19.02, 28.87] | | 24.17 | 2.7 | [18.93, 29.40] |
|  |  | PP2 | Stem *Pentameris* | 17.47 | [12.99, 23.14] | | 17.51 | 2.6 | [12.41, 22.60] |
| Poa | *Ehrharta* | PE | Split Bambusoideae+Pooideae-Ehrhartoideae | 33.55 | [28.49, 39.73] | | 34.08 | 2.9 | [28.30, 39.86] |
| Poa | *Elegia-Thamnochortus* | PET1 | Crown *Baloskion* | 60.07 | [57.83, 64.24] | | 60.07 | 1.9 | [56.35, 63.79] |
| Pro | *Protea* | PP1 | Crown Proteoideae II (B) | 75.66 | [71.36, 80.20] | | 75.66 | 2.2 | [71.25, 80.07] |
|  |  | PP2 | Crown Proteeae (67) | 30.25 | [16.75, 46.38] | | 30.25 | 7.6 | [15.35, 45.15] |
|  |  | PP3 | Crown Leucadendrinae (59) | 28.11 | [18.57, 38.27] | | 28.11 | 5.1 | [18.11, 38.11] |
|  |  | PP4 | Crown *Protea* (66) | 12.32 | [5.06, 20.56] | | 12.32 | 3.9 | [4.56, 20.08] |
| Pro | *Leucadendron* | PL1 | Crown Leucadendreae (61) | 44.52 | [31.96, 58.26] | | 44.52 | 6.8 | [31.17, 57.87] |
|  |  | PL2 | Crown *Adenanthos*+Leucadendrinae (60) | 33.91 | [22.84, 45.59] | | 33.91 | 5.9 | [22.19, 45.63] |
|  |  | PL3 | Crown Leucadendrinae (59) | 28.11 | [18.57, 38.27] | | 28.11 | 5.1 | [18.11, 38.11] |
|  |  | PL4 | Crown *Serruria* (54) | 16.35 | [10.23, 22.37] | | 16.35 | 3.1 | [10.27, 22.43] |

For each species-level dating analysis (column 2), the higher-level analysis used as a calibration reference is indicated in column 1 (Ast = Asteraceae; Asp = Asparagales; Orc = Orchidaceae; Poa = Poales; Pro = Proteaceae), while the calibration nodes are identified in columns 3 and 4. Column 3 provides the node labels used in Figs A-L in S1 File. For calibrations done using existing higher-level analyses, the original node labels, where supplied, are also provided (column 4, in parentheses). Columns 5 and 6 indicate the median node heights and associated 95% HPDs derived from the higher-level analysis, while columns 7 and 8 indicate the means and standard deviations (s.d.) specified for the priors on the species-level analyses, and column 39 the 95% CIs associated with these settings.
