# Supplementary material for: Dated Plant Phylogenies Resolve Neogene Climate and Landscape Evolution in the Cape Floristic Region
Source: PLoS One. 2015 Sep 30;10(9):e0137847. doi: 10.1371/journal.pone.0137847 (PMC4589284; doi:10.1371/journal.pone.0137847)
Supplement: S1 File — (ZIP) [file pone.0137847.s001.zip › Supporting Information 1_S1/Table C.docx]

**Table C.** **Posterior estimates obtained from the molecular dating estimates.**

| Group | UCLD | | Covariance | |
| --- | --- | --- | --- | --- |
|  | stdev (σ) | 95% CI | mean | 95% CI |
| Orchidaceae | 0.4787 | [0.3906, 0.5714] | 0.1057 | [-0.0440, 0.2534] |
| Poales | 0.6620 | [0.5672, 0.7629] | 0.0409 | [-0.0799, 0.1648] |
| Arctotidinae | 0.623 | [0.3925, 0.8579] | 0.0384 | [-0.1075, 0.1946] |
| *Stoebe* | 0.5773 | [0.3588, 0.7969] | 0.0474 | [-0.1490, 0.2427] |
| Coryciinae | 0.4195 | [0.3389, 0.5010] | 0.0049 | [-0.1483, 0.1494] |
| *Satyrium* | 0.6814 | [0.5534, 0.8127] | 0.0639 | [-0.0631, 0.1890] |
| Danthonioideae | 0.6975 | [0.5964, 0.8055] | 0.0754 | [-0.0282, 0.1954] |
| *Ehrharta* | 0.6714 | [0.2982, 1.0874] | 0.0308 | [-0.2327, 0.3124] |
| *Elegia-Thamnochortus* | 0.3670 | [0.2473, 0.4499] | 0.0065 | [-0.1277, 0.1423] |
| *Protea* | 0.8436 | [0.7096, 0.9884] | 0.0591 | [-0.0791, 0.2076] |
| *Leucadendron* | 0.8496 | [0.5505, 1.1794] | 0.0398 | [-0.1409, 0.2316] |
| *Moraea* | 0.6522 | [0.5514, 0.7568] | 0.0087 | [-0.0892, 0.1081] |

Posterior estimates (mean and 95% confidence interval) of (i) the standard deviation of the UCLD clock rate, and (ii) the covariance parameter, yielded by the BEAST analyses.
