# Supplementary material for: Dated Plant Phylogenies Resolve Neogene Climate and Landscape Evolution in the Cape Floristic Region
Source: PLoS One. 2015 Sep 30;10(9):e0137847. doi: 10.1371/journal.pone.0137847 (PMC4589284; doi:10.1371/journal.pone.0137847)
Supplement: S1 File — (ZIP) [file pone.0137847.s001.zip › Supporting Information 1_S1/Table D.docx]

**Table D.** **Comparison of molecular age estimates obtained in this study with those obtained in previous dating analyses.**

| Group | Method | N | Discrepancy score  (%; mean [range]) | Source |
| --- | --- | --- | --- | --- |
| Orchidaceae | PL* | 7 | -22.8 [-6.8, -31.7] | [5] |
| Orchidaceae | NPRS* | 7 | -27.2 [-5.7, -49.4] | [5] |
| Orchidaceae | BEAST* | 7 | -13.6 [-2.6, -21.8] | [6] |
| Poales | NPRS* | 5 | -24.3 [-10.6, -36.4] | [7] |
| Poales (Poaceae) | Multidivtime* | 2 | -25.6 [-21.0, -30.2] | [8] |
| Poales (Poaceae) | Multidivtime* | 5 | -18.1 [5.1, -37.4] | [9] |
| Poales (Poaceae) | BEAST* | 10 | -30.2 [35.3, -55.0] | [10] |
| Arctotidinae | BEAST‡ | 5 | 24.5 [25.8, -29.5] | [11] |
| *Stoebe* | BEAST†§ | 4 | -37.2 [-27.8, -46.2] | [12] |
| *Ehrharta* | NPRS† | 2 | -59.7 [-56.5, -62.9] | [13] |
| *Elegia-Thamnochortus* | Multidivtime† | 8 | -13.1 [-5.8, -19.4] | [14] |
| *Moraea* | BEAST† | 6 | 4.3 [-4.5, 10.1] | [15] |
| *Protea* | BEAST† | 6 | 10.0 [7.8, -24.3] | [16] |

Each discrepancy is expressed as the difference between published and current age estimates, expressed as a percentage of the former. Negative discrepancy scores indicate that the published age estimate was older (see also Fig P in S1 File). Symbols in column 2 indicate the calibration method (* = fossil, † = secondary calibration, ‡ = external rates, § = biogeographic constraint). The number of nodes underpinning each comparison is indicated (N).
