# Supplementary material for: Dated Plant Phylogenies Resolve Neogene Climate and Landscape Evolution in the Cape Floristic Region
Source: PLoS One. 2015 Sep 30;10(9):e0137847. doi: 10.1371/journal.pone.0137847 (PMC4589284; doi:10.1371/journal.pone.0137847)
Supplement: S1 File — (ZIP) [file pone.0137847.s001.zip › Supporting Information 1_S1/Table E.docx]

**Table E. Estimated times (Ma) of earliest occupation of alternative climatic and substratum habitats within each group.**

| Group | PDQ | | PCV | | Substratum | | | | |
| --- | --- | --- | --- | --- | --- | --- | --- | --- | --- |
|  | >75 mm | <75 mm | <60% | >60% | Qua | Sha | Cal | All | Low |
| Arctotidinae | 8.9 [5.2, 13.2] | 9.7 [5.7, 14.5] | 11.9 [7.3, 17.3] | 1.7 [0.8, 3.0] | 5.7 [3.2, 9.0] |  |  | 3.2 [1.3, 6.1] |  |
| *Disperis* | 18.4 [14.0, 23.1] | 6.5 [4.3, 9.1] | 18.4 [14.0, 23.1] | 0.3 [0.1, 0.6] | 18.4 [14.0, 23.1] | 5.5 [3.6, 7.8] |  |  |  |
| *Ehrharta* | 13.3 [7.6, 21.9] | 4.7 [2.1, 8.9] | 13.3 [7.6, 21.9] | 2.1 [0.8, 4.4] | 13.3 [7.6, 21.9] | 4.7 [2.1, 8.9] |  |  |  |
| *Elegia-Thamnochortus* | 28.3 [20.8, 36.7] | 15.7 [10.8, 21.9] | 28.3 [20.8, 36.7] | 13.5 [9.6, 18.0] | 28.3 [20.8, 36.7] | 11.7 [8.3, 15.8] | 6.9 [4.1, 10.5] | 1.7 [0.8, 3.1] | 16.6*[12.2, 21.8] |
| *Leucadendron* | 21.2 [14.2, 28.3] | 18.9 [12.7, 25.6] | 21.2 [14.2, 28.3] | 6.5 [2.5,11.6] | 21.2 [14.2, 28.3] | 5.6 [1.6, 11.2] | 6.5 [2.5,11.6] | 2.0 [0.3, 5.3] | 1.6 [0.2, 4.0] |
| *Moraea* | 4.1 [2.1, 6.6] | 17.5 [12.8,22.7] | 17.5 [12.8,22.7] | 7.6 [4.1, 11.7] | 17.5 [12.8,22.7] | 5.0 [3.1, 7.1] | 4.0 [2.5,5.8] | 1.7[0.8,2.7] |  |
| *Pentameris* CP | 14.5 [10.9, 18.0] | 11.7 [ 8.6, 15.0] | 14.5 [ 10.9, 18.0] | 7.6 [5.6,10.1] | 14.5 [ 10.9, 18.0] | 4.3 [2.0, 7.6] | 5.1 [3.4, 6.9] |  | 2.8 [0.9,5.4] |
| *Pentameris* NR | 14.5 [10.9, 18.0] | 11.7 [ 8.6, 15.0] | 14.5 [ 10.9, 18.0] | 7.6 [5.6,10.1] | 14.5 [ 10.9, 18.0] | 4.3 [2.0, 7.6] | 5.1 [3.4, 6.9] |  | 2.8 [0.9,5.4] |
| *Protea* | 19.4 [14.7, 24.3] | 15.1 [10.8, 19.5] | 19.4 [14.7, 24.3] | 7.0 [3.9, 10.7] | 19.4 [14.7, 24.3] | 11.8 [8.0, 16.4] | 7.0 [3.9, 10.7] | 3.4 [1.0, 7.4] | 4.7 [2.1, 7.6] |
| *Pterygodium* | 13.9 [9.4, 18.8] | 18.8 [14.2, 23.9] | 18.8 [14.2, 23.9] | 6.5 [4.5, 8.9] | 18.8 [14.2, 23.9] | 1.1 [0.4, 2.1] |  |  | 3.1 [1.7, 4.8] |
| *Satyrium* | 10.3 [6.8,14.3] | 4.1 [2.3, 6.6] | 10.3 [6.8,14.3] |  | 10.3 [6.8,14.3] |  | 6.4 [3.8, 9.4] |  |  |
| *Stoebe* | 2.9 [1.1, 6.7] | 5.0 [2.6, 8.2] | 5.0 [2.6, 8.2] | 3.6 [1.9, 5.8] | 5.0 [2.6, 8.2] | 1.5 [0.7, 2.6] | 1.4 [0.7, 2.3] | 1.9 [0.7, 3.9] | 1.8 [0.8, 2.9] |
| *Tribolium* CP | 1.9 [0.5, 4.0] | 6.9 [4.7, 9.4] | 6.9 [4.7, 9.4] | 6.9 [4.7, 9.4] | 1.9 [0.5, 4.0] | 5.1 [3.3, 7.2] | 6.2 [4.3, 8.7] |  |  |
| *Tribolium* NR | 1.9 [0.5, 4.0] | 6.9 [4.7, 9.4] | 6.9 [4.7, 9.4] | 6.9 [4.7, 9.4] | 1.9 [0.5, 4.0] | 2.4 [1.1, 4.0] | 6.9 [4.7, 9.4] |  |  |
| Mean | 13.1 | 11.2 | 15.5 | 5.7 | 14.5 | 6.1 | 5.4 | 2.3 | 5.1 (2.9) |
| Median | 13.6 | 10.7 | 16.0 | 6.5 | 16.0 | 5.1 | 6.3 | 2.8 | 2.8 |

The 95% HPD confidence intervals for each age estimate are given in square brackets. For *Pentameris* and *Tribolium*, the results of reconstructions done using both the plastid (CP) and nuclear (NR) trees are provided. Means and median times of occupation of each habitat state are provided which, in all instances, incorporate the chloroplast-based results for *Pentameris* and *Tribolium*. The mean time of earliest occupation of lowland sands was determined with the outlying value for *Elegia-Thamnochortus* (*) both included and excluded (brackets). Abbreviations for substrate type as follows: Qua = quartzite, Sha = shale, Cal = calcrete, All = alluvial, Low = lowland sands.
