# Supplementary material for: Dated Plant Phylogenies Resolve Neogene Climate and Landscape Evolution in the Cape Floristic Region
Source: PLoS One. 2015 Sep 30;10(9):e0137847. doi: 10.1371/journal.pone.0137847 (PMC4589284; doi:10.1371/journal.pone.0137847)
Supplement: S1 File — (ZIP) [file pone.0137847.s001.zip › Supporting Information 1_S1/Table F.docx]

**Table F. Diversification statistics for the 12 study lineages.**

| Group | Diversification model | | | | |
| --- | --- | --- | --- | --- | --- |
|  | model | ΔAICrc | *p_1_* | *p_2_* | *t* |
| Arctotidinae | Y2R ↑ | 0.980 | 0.1534 | 0.3637 | 3.61 |
| *Disperis* | BD | -0.633 | 0.0835 | NA | NA |
| *Ehrharta* | Y2R ↑ | 0.098 | 0.0776 | 0.2916 | 4.75 |
| *Elegia-Thamnochortus* | BD | -0.731 | 0.1362 | NA | NA |
| *Leucadendron* | BD | -1.462 | 0.1818 | NA | NA |
| *Moraea* | YR2 ↓ | 3.411 | 0.2304 | 0.0805 | 0.63 |
| *Pentameris* | Y2R ↓ | 4.633 | 0.2933 | 0.1355 | 1.37 |
| *Protea* | Y2R ↓ | 17.066 | 0.1894 | 0.0263 | 1.66 |
| *Pterygodium* | BD | -1.061 | 0.1480 | NA | NA |
| *Satyrium* | DDL | 0.438 | 0.4027 | 16.435 | NA |
| *Stoebe* | Y2R ↓ | 13.943 | 0.8981 | 0.2597 | 1.02 |
| *Tribolium* | DDL | 2.609 | 0.6959 | 14.7925 | NA |

Columns 2-6 present the attributes of the models [4] that were identified as best describing patterns of lineage accumulation. Column 2 indicates the model identified as optimal (Y2R = Yule two-rate; BD = constant-rate, birth-death; DDL = logistic density dependent model), column 3 the AIC score associated with that model, and columns 4-6 the parameter estimates. For the birth-death model, p1 is the net diversification rate. For the Yule two-rate model, p1 and p2 are the diversification rates prior to and following a rate switch taking place at time t. For the DDL model, p1 is the initial diversification rate and p2 is the K or ‘carrying capacity’ parameter.
