# Supplementary figures and images for: Dated Plant Phylogenies Resolve Neogene Climate and Landscape Evolution in the Cape Floristic Region
Source: PLoS One. 2015 Sep 30;10(9):e0137847. doi: 10.1371/journal.pone.0137847 (PMC4589284; doi:10.1371/journal.pone.0137847)

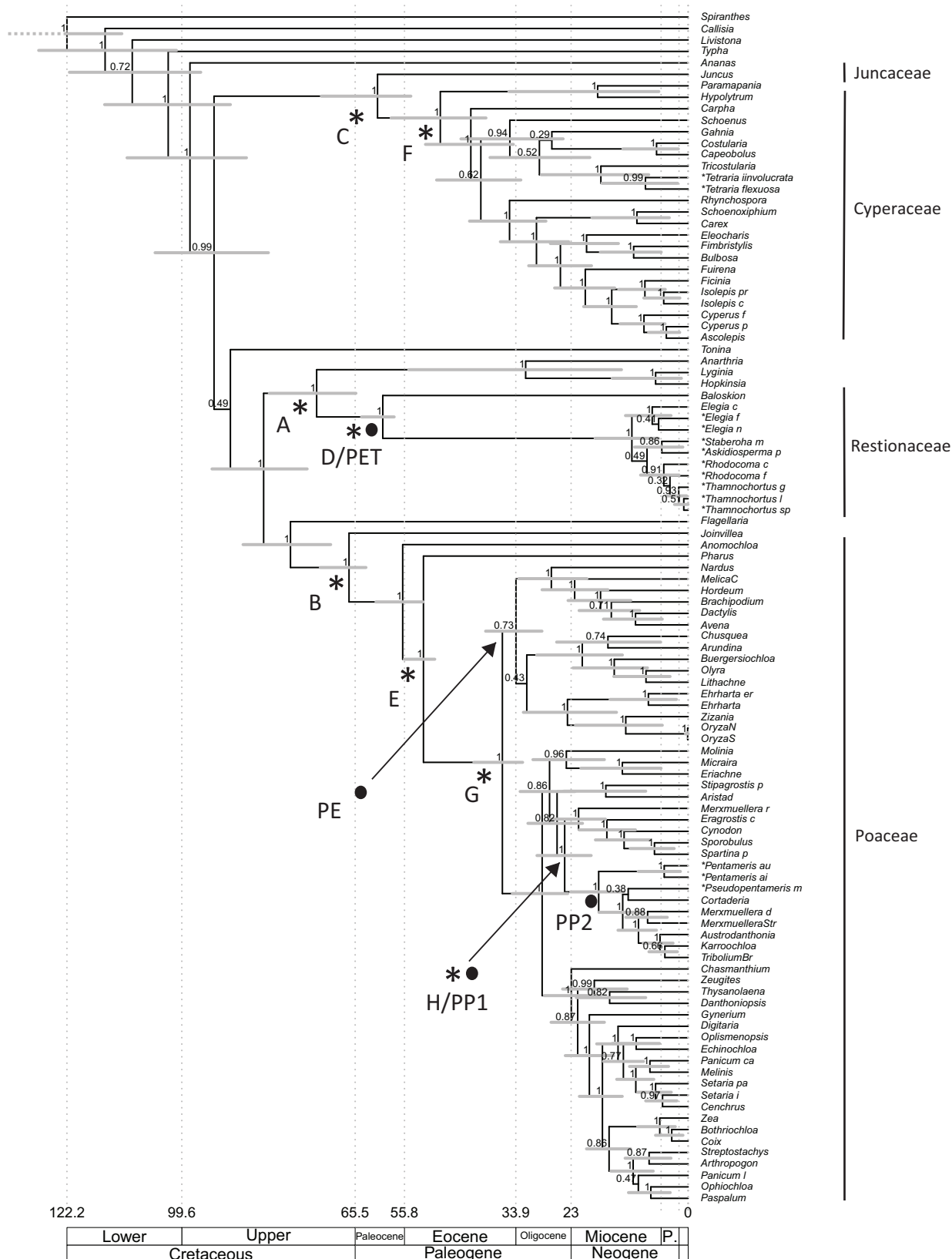

Supplement: S1 File — (ZIP) [file pone.0137847.s001.zip › Supporting Information 1_S1/Fig B.pdf]
